# Integrated Item Response Theory Modeling of Multiple Patient-Reported Outcomes Assessing Lower Urinary Tract Symptoms Associated with Benign Prostatic Hyperplasia

**DOI:** 10.1208/s12248-020-00484-7

**Published:** 2020-07-29

**Authors:** Yassine Kamal Lyauk, Trine Meldgaard Lund, Andrew C. Hooker, Mats O. Karlsson, Daniël M. Jonker

**Affiliations:** 1 Translational Medicine, Ferring Pharmaceuticals A/S, Copenhagen, Denmark; 2 Department of Drug Design and Pharmacology, University of Copenhagen, Copenhagen, Denmark; 3 Department of Pharmaceutical Biosciences, Uppsala University, Uppsala, Sweden

**Keywords:** BPH, BPH Impact Index, Item Response Theory, International Prostate Symptom Score, LUTS, Quality of Life

## Abstract

In clinical trials within lower urinary tract symptoms due to benign prostatic hyperplasia (BPH-LUTS), the International Prostate Symptom Score (IPSS) is commonly the primary efficacy outcome while the Quality of Life (QoL) score and the BPH Impact Index (BII) are common secondary efficacy markers. The current study aimed to characterize BPH-LUTS progression using responses to the IPSS, the QoL, and the BII in an integrated item response theory (IRT) framework and assess the Fisher information of each scale. The power of this approach to detect a drug effect was compared with an IRT approach considering only IPSS responses. A unidimensional and a bidimensional pharmacometric IRT model, based on item-level IPSS responses in a clinical trial with 403 patients, were extended by incorporating patients’ QoL and summary BII scores over the 6-month trial period. In the developed unidimensional integrated model, the QoL score was found to be the most informative, representing 17% of the total Fisher information, while the combined information content of the seven IPSS items represented 70.6%. In the bidimensional model, “storage” and both storage and “voiding” disability drove QoL and summary BII responses, respectively. Sample size reduction of 16% to detect a drug effect at 80% power was obtained with the unidimensional integrated IRT model compared with its counterpart IPSS IRT model. This study shows that utilizing the information content across the IPSS, QoL, and BII scales in an integrated IRT framework results in a modest but meaningful increase in power to detect a drug effect.

**Electronic supplementary material:**

The online version of this article (10.1208/s12248-020-00484-7) contains supplementary material, which is available to authorized users.

## INTRODUCTION

As the prostate enlarges with age, older men may suffer from the obstruction of the prostatic urethra and deterioration of the urethral sphincter function (1). This condition is known as benign prostate hyperplasia (BPH) and is estimated to affect 50% of the male population by age 60 years (2,3). Lower urinary tract symptoms (LUTS) often develop due to BPH and are thought to stem from a combination of both static and dynamic factors of BPH as well as the bladder’s response to outflow obstruction (4,5). The prevalence of BPH-LUTS is similar across different countries (6–11) and can hence be considered a medical condition with a substantial impact on public health globally speaking.

To assess BPH-LUTS, which, in addition to urinary function, may impact patients’ general well-being as well as different facets of their everyday life, three validated, disease-specific, patient-reported outcomes (PROs) are conventionally used. The International Prostate Symptom Score (IPSS) (also called as the American Urological Association Symptom index) (12) is the most widely used PRO within BPH-LUTS (13,14) and consists of seven items that each can be rated from zero to five. IPSS voiding items describe the severity of a feeling of incomplete emptying of the bladder following urination, urination intermittency, the urgency to urinate, the weakness of the urinary stream, and straining during urination. IPSS storage items describe urination frequency, the urgency to urinate, and nocturia (15). Current versions of the IPSS questionnaire include an additional question following the seven IPSS items, known as the Quality of Life (QoL) or “bother” question (16). The QoL question assesses a patient’s perception of his current health state by asking how he would feel if he were to spend the rest of his life with his urinary condition. It can be rated from zero to six, zero corresponding to “Delighted” and six to “Terrible.” Lastly, the BPH Impact Index (BII) (17) is a four-item questionnaire that assesses the physical discomfort associated with urinary problems, the degree of worrying regarding health due to urinary problems, the perception of overall bother associated with urination, and the hindering of performance of desired activities due to urinary problems. Three of the BII items are rated from zero to three while one is rated from zero to four, resulting in a summary BII ranging from zero to 13.

In clinical trials investigating treatment of BPH-LUTS, the summary IPSS is conventionally specified as the primary efficacy outcome measure, while the QoL and summary BII are specified as secondary efficacy markers (13). These three scales may contribute different insights into BPH-LUTS, and it may hence be of value to regard the information of these scales jointly rather than separately to more precisely determine the severity of BPH-LUTS in patients. Item Response Theory (IRT) models can be used to incorporate information from multiple PROs to assess the impact of a given disease, giving higher weight to more sensitive PROs, while still capturing information from less sensitive ones. As its name suggests, IRT utilizes the item-level responses in questionnaires to estimate an individual’s level of disability (e.g., underlying BPH-LUTS), the sensitivity of each item to change in disability, and the thresholds of item scores along the disability scale. Because IRT uses item-level data and quantifies item sensitivity, an integrated IRT model regarding information from the IPSS, QoL, and BII jointly may allow for a powerful approach for assessing BPH-LUTS and detecting drug effects. IRT analyses combining information from different scales have been performed within the therapeutic areas of neonatal pain (18,19) and migraine (20), but, to date, not within BPH-LUTS.

Building on a recent pharmacometric IRT model based on item-level IPSS data in a clinical trial with the GnRH antagonist degarelix (21), the current study aims to characterize BPH-LUTS progression by joint analysis of item-level IPSS, the QoL score, and BII data in an integrated IRT framework while assessing the informativeness of each scale. The power of this integrated BPH-LUTS IRT model to detect a drug effect will be compared with the longitudinal IRT model considering only IPSS responses.

## METHODS

### Data

Data from Ferring Pharmaceuticals A/S trial CS36 (NCT00947882) was utilized in the current work, which was also used for the development of a previous longitudinal IPSS IRT model (21). CS36 was a Phase II placebo-controlled, double-blind, parallel-group, randomized dose-finding study, where a single subcutaneous injection of either 10, 20, or 30 mg of the GnRH antagonist degarelix 40 mg/mL solution was administered to patients. The trial enrolled 403 patients with an IPSS ≥ 13 and a QoL score ≥ 3 at the screening visit 2 weeks before dosing at baseline. Over the 6-month trial period, eight visits were planned (a baseline visit and 14 days and 1, 2, 3, 4, 5, and 6 months after dosing). Item-level IPSS and the QoL score were assessed at each of these visits, while the summary BII (BII_summary_) was assessed at three visits (baseline visit and 3 and 6 months after dosing). Due to the unavailability of item-level BII responses, the BII_summary_ was considered an item with 14 possible categories.

### Item Response Theory Analysis

Psychometrically, regarding the IPSS as either unidimensional or bidimensional is valid based on multiple studies within BPH-LUTS (21–23). Building on this prior knowledge, both unidimensional and multidimensional integrated IRT approaches were investigated in the current work.

#### Unidimensional Item Response Theory Modeling

A unidimensional IRT model was first fit to the data assuming a single latent construct driving patients’ responses to the IPSS, QoL, and BII_summary_. Data from all individuals and all visits were used to estimate the item characteristic curves (ICCs) (24–27) (termed the *IDVIS* approach (21)). The reference baseline latent variable distribution(s) was fixed to standard normal distributions *N*(0,1), and post-baseline shift parameters were estimated to account for differences in the distribution of latent disability following intervention (placebo or treatment) while assuming that ICCs are constant (24–27).

Each BPH-LUTS measure contains at least six item-response categories (zero to five for the IPSS items, zero to six for the QoL score, and zero to 13 for the BII_summary_ item). The probability of a patient answering at least *k* based on his latent disability was described using a graded response model (28):

$$P\left({Y}_{ij}\ge k\right)=\frac{e^{a_{j\kern0.5em }\left({\psi}_i-{b}_{j\cdotp k}\right)}}{1+{e}^{a_{j\kern0.5em }\left({\psi}_i-{b}_{j\cdotp k}\right)}}$$

where *a*_*j*_ represents the slope/discrimination parameter of item *j*, *ψ*_*i*_ the latent disability of patient *i*, and *b*_*j*_ the difficulty/location parameter of item *j* for category *k*. Cumulative probabilities for an item with a score of maximum *X* were modeled as:

$${\displaystyle \begin{array}{c}P\left({Y}_{ij}=0\right)=1-P\left({Y}_{ij}\ge 1\right)\\ {}P\left({Y}_{ij}=k\right)=P\left({Y}_{ij}\ge k\right)-P\left({Y}_{ij}\ge k+1\right)\\ {}P\left({Y}_{ij}=X\right)=P\left({Y}_{ij}\ge X\right)\end{array}}$$

where *X* is five for each of the seven IPSS items, six for the QoL score, and 13 for the BII_summary_. Following ICC estimation, the original individual assignment was reconciled with the data, and the longitudinal model was combined with the IRT ICC model to describe the relationship between changes in disability over time and response probability.

#### Multidimensional Item Response Theory Modeling

Factor analysis is an established statistical method for informing the item structure of IRT models (29). It aims to explain the correlation between items by assuming that one or more latent variables (factors) steer responses to these items. The factor loadings indicate the covariance between each item and the factor(s) and allow for dimensionality assessment. Factor analysis may be exploratory or confirmatory in nature: the former does not pre-specify the number of factors to explore while the latter does. Building on the bidimensional IRT model that regarded only item-level IPSS (21), the item structure of an integrated multidimensional IRT model was explored through confirmatory factor analysis using two and three dimensions, respectively. Given that a minimum of three items per latent variable is required to preserve IRT model identification, no more than three latent variables were explored in the current analysis. Varimax orthogonal rotation (30) was used as the rotation method during factor analysis. If an item was found to not be predominantly correlated with a single factor, a compensatory graded response model for polytomous data (31) was implemented to allow multiple latent variables to affect the probability of responses for this item. In the compensatory graded response model, the probability of a patient answering at least *k* for item *j* is:

$${\displaystyle \begin{array}{c}P\left({Y}_{ij}\ge k\right)=\frac{e^{\left({a}_{m,j\ast }{\psi}_m-{B}_{k,j}\right)}}{1+{e}^{\left({a}_{m,j\ast }{\psi}_m-{B}_{k,j}\right)}}\\ {}\mathrm{with}\ {a}_{m,j}\ast {\psi}_m={a}_{1,j}\ast {\psi}_1+{a}_{2,j}\ast {\psi}_2+\dots +{a}_{m,j}\ast {\psi}_m\end{array}}$$

where *m* is the number of latent variables, *ψ*_*m*_ the vector of latent disability estimates for patient *i*, *a*_*j*_ the item-specific discrimination parameters associated with each latent disability, and *B*_*k*_ the overall difficulty of the item response category *k*.

### Calculation of Fisher Information Content

The Fisher information content of each item in the unidimensional integrated IRT model was calculated as minus the expectation of the second derivative of the log-likelihood. The sensitivity of each item over the current study’s disability range was visualized through their information functions. Ranking of individual items was performed according to the amount of information they contained relative to the total information. This was achieved by calculating the area under the curve for each item divided by the sum of all areas under the curve. The unidimensional IRT model was used for calculation of Fisher Information content as it allows for comparison of the information content among all included items based on the same common latent scale.

### Longitudinal Modeling and Covariate Analysis

Longitudinal integrated IRT model development was similar to that previously reported for longitudinal IRT modeling based on IPSS data and readers are referred to Lyauk *et al.* (21) for more details. Briefly, data from patients receiving placebo treatment were first modeled to describe the placebo effect, and subsequently, data from patients that received degarelix were added to the dataset to describe the drug effect. Following structural longitudinal model development, baseline demographics (age, weight, and body mass index), baseline physiological disease-specific measures (total prostate volume, serum testosterone, prostate-specific antigen, average flow rate, flow time including time to maximum flow, maximum urine flow, post-void residual volume, voiding time, and voiding volume), and study site region (North America or Europe) were investigated as covariates. For this purpose, a stepwise search (SCM) at a significance level of 0.01 in the forward inclusion step and 0.001 in the backward elimination step. A multiplicative covariate model was used for all parameters except those where the typical value was expected to be zero or close to zero, such as baseline disability. If this was the case, an additive covariate model was used.

### Software

NONMEM version 7.4.3 with the Laplacian method was used for ICC estimation and longitudinal IRT modeling. Perl-Speaks-NONMEM (32) (PsN) version 4.9.0 was used for simulation-based model diagnostics, and the mirt package version 1.31 in R 3.6.0 (33) was used for factor analyses and to obtain initial estimates for ICC estimation in NONMEM.

### Model Evaluation and Diagnostics

The goodness of fit of the ICCs was assessed using the Empirical Bayes Estimate-based (26), as well as the sampling-based (21), cross-validated generalized additive model (GAM) cubic spline smooth. Longitudinal model selection was based primarily on the change in objective function (OFV) and secondly on assessment of visual predictive checks (VPCs). For nested models, a difference in OFV corresponding to a prespecified significance level (*α* = 0.05 for everything but covariate analysis) was assumed statistically significant assuming a *χ*^2^ distribution. For non-nested models, the Akaike Information Criterion (AIC) was used. In longitudinal IRT modeling, fixing the ICC parameters to the values obtained in the ICC estimation step while estimating the longitudinal parameters and simultaneously estimating the ICCs and longitudinal parameters, respectively, was investigated in terms of OFV reduction. VPCs were used to assess the adequacy of the developed longitudinal models using 200 samples.

### Power Calculations

Power to detect a drug effect was determined by way of clinical trial simulations with the respective final integrated IRT models. The stochastic simulation and estimation (sse) procedure in Perl-Speaks-NONMEM PsN (32) was used specifying 1000 simulated data sets at four different sample sizes while respecting the treatment to placebo allocation ratio in the original CS36 data set. An initial Monte Carlo Mapped Power (MCMP) procedure (34) informed the determination of the sizes of these four data sets. No missing item responses, as well as no dropout, was assumed in the simulations. A threshold of 3.84 (*p* = 0.05) was used to identify significant reductions in OFV between the respective full (estimating a drug effect) and reduced (not estimating a drug effect) models. Type I error was investigated by simulating 1000 data sets under each sample size from the integrated IRT model with no drug effect. The proportion of subsequent estimations where the drug effect was identified as significant determined the type I error rate.

## RESULTS

**Fig. 1:**
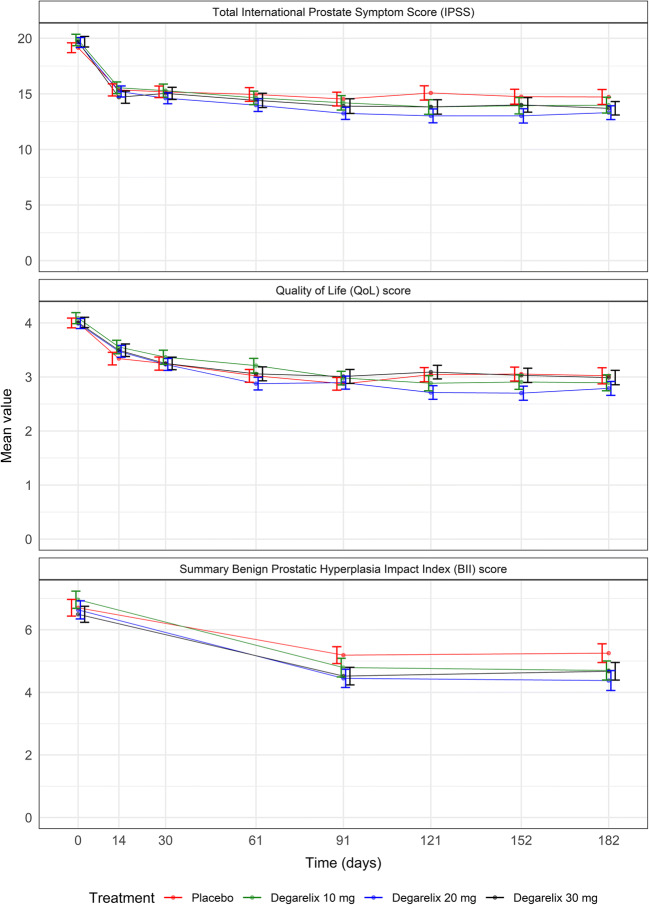
Time course of the mean total International Prostate Symptom Score, Quality of Life score, and summary Benign Prostatic Hyperplasia Impact Index. Standard errors are indicated as error bars

The CS36 trial enrolled 403 patients, of which 369 completed the six-month treatment period. The baseline patient population characteristics have been presented elsewhere (21). A total of 21,836 item-level IPSS, 3119 QoL scores, and 1116 BII_summary_ observations over the 6-month trial period were available for analysis in the current work. Figure 1 shows the mean time course for the total IPSS, the QoL score, and the BII, respectively, in the CS36 trial. A marked drop in mean score was observed for all treatment arms on each BPH-LUTS scale and no dose-response relationship was apparent on any of the three scales. The Supplemental Material contains further details on the distribution of responses in each BPH-LUTS scale.

### Unidimensional Integrated Item Response Theory Modeling

**Fig. 2:**
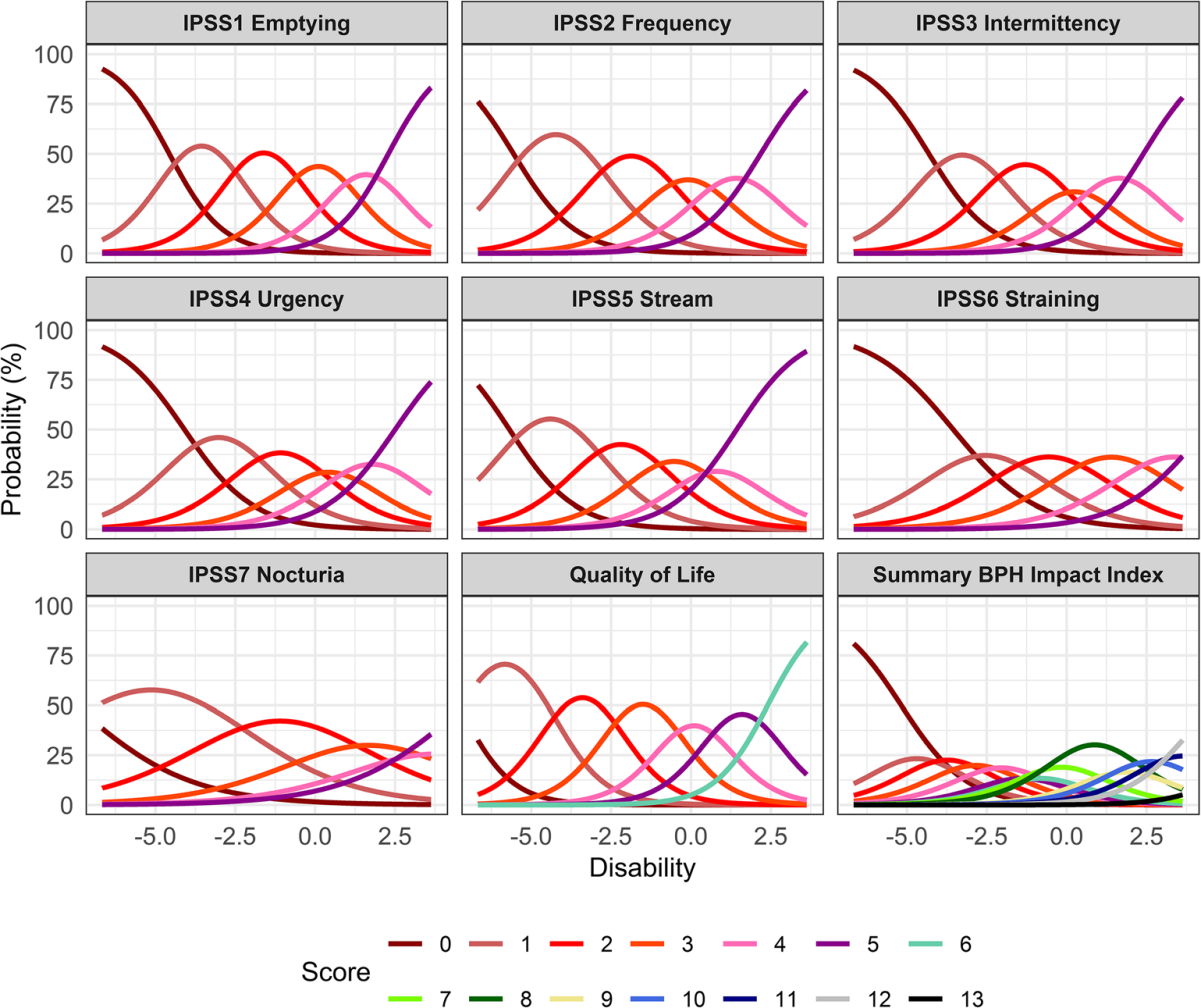
Item characteristic curves in the unidimensional integrated item response theory model. IPSS, International Prostate Symptom Score; BPH, Benign Prostatic Hyperplasia

The item characteristic curves (ICCs) for the seven IPSS items, the QoL item, and the BII_summary_ item in the unidimensional integrated BPH-LUTS IRT model are shown in Fig. 2**,** and the corresponding ICC parameter estimates are shown in Table I. The latter were overall estimated with low uncertainty, although higher uncertainty was observed for BII_summary_ difficulty parameters. The discrimination parameter value was lowest for the *Nocturia* IPSS item (0.55) and highest for the QoL item (1.22), indicating that they respectively have the lowest and highest sensitivity to change in disability. Adequate fit of the ICCs was observed with GAM diagnostics and these are shown in the Supplemental Material.

**Table I:** Item Characteristic Curve Parameter Estimates in the Integrated Unidimensional Lower Urinary Tract Symptoms Due to Benign Prostatic Hyperplasia (BPH) Item Response Theory Model

Parameter	Estimate	Relative standard error (%)
a_IPSS1_	1.19	7.3
b_IPSS1,1_	− 4.56	6
b_IPSS1,2_	2.02	7.3
b_IPSS1,3_	1.86	6.8
b_IPSS1,4_	1.57	7
b_IPSS1,5_	1.4	8.1
a_IPSS2_	1.04	6.8
b_IPSS2,1_	− 5.55	6
b_IPSS2,2_	2.65	7.3
b_IPSS2,3_	2.06	6.7
b_IPSS2,4_	1.49	7
b_IPSS2,5_	1.53	7.5
a_IPSS3_	1.04	7.5
b_IPSS3,1_	− 4.31	6
b_IPSS3,2_	2.09	7.4
b_IPSS3,3_	1.85	7.1
b_IPSS3,4_	1.23	7.5
b_IPSS3,5_	1.53	8.2
a_IPSS4_	0.929	6.9
b_IPSS4,1_	− 4.09	5.8
b_IPSS4,2_	2.14	7
b_IPSS4,3_	1.74	6.8
b_IPSS4,4_	1.26	7.4
b_IPSS4,5_	1.45	7.9
a_IPSS5_	0.972	7
b_IPSS5,1_	− 5.68	6.2
b_IPSS5,2_	2.56	7.7
b_IPSS5,3_	1.87	7
b_IPSS5,4_	1.45	7.1
b_IPSS5,5_	1.23	7.7
a_IPSS6_	0.774	8.1
b_IPSS6,1_	− 3.55	6.3
b_IPSS6,2_	2.01	8
b_IPSS6,3_	1.96	7.9
b_IPSS6,4_	1.96	8.6
b_IPSS6,5_	1.96	10.3
a_IPSS7_	0.549	7.6
b_IPSS7,1_	− 7.52	6.8
b_IPSS7,2_	4.79	7.9
b_IPSS7,3_	3.28	7.4
b_IPSS7,4_	2.26	8.1
b_IPSS7,5_	1.91	9.9
a_QoL_	1.22	6.4
b_QoL1_	− 7.26	6.3
b_QoL,2_	2.88	9.4
b_QoL,3_	1.97	6.8
b_QoL,4_	1.82	6.4
b_QoL,5_	1.38	6.7
b_QoL,6_	1.61	7.5
a_BII_	0.975	7.9
b_BII,1_	− 5.18	6.6
b_BII,2_	0.974	13.8
b_BII,3_	0.943	11.8
b_BII,4_	0.828	11.1
b_BII,5_	0.775	10.4
b_BII,6_	0.549	11.4
b_BII,7_	0.552	11.1
b_BII,8_	0.789	10
b_BII,9_	1.28	9.7
b_BII,10_	0.685	14.5
b_BII,11_	0.91	15.5
b_BII,12_	1.03	20.1
b_BII,13_	2.48	28.8
Post-baseline disability variance	2.59	6.3
Post-baseline disability mean	− 1.53	5.9

Relative standard error was calculated as 100 * (standard error of estimate / estimate). The typical value of *η*-shrinkage was 8.2%

*a*, discrimination parameters; *b*, difficulty parameters for each score using the delta method (e.g., B_IPSS1,2_ = b_IPSS1,1_ + b_IPSS1,2_); *IPSS*, International Prostate Symptom Score; *QoL*, Quality of Life; *BII*, Summary BPH Impact Index; *IPSS1*, incomplete emptying; *IPSS2*, frequency; *IPSS3*, intermittency; *IPSS4*, urgency; *IPSS5*, weak stream; *IPSS6*, straining; *IPSS7*, nocturia

**Fig. 3:**
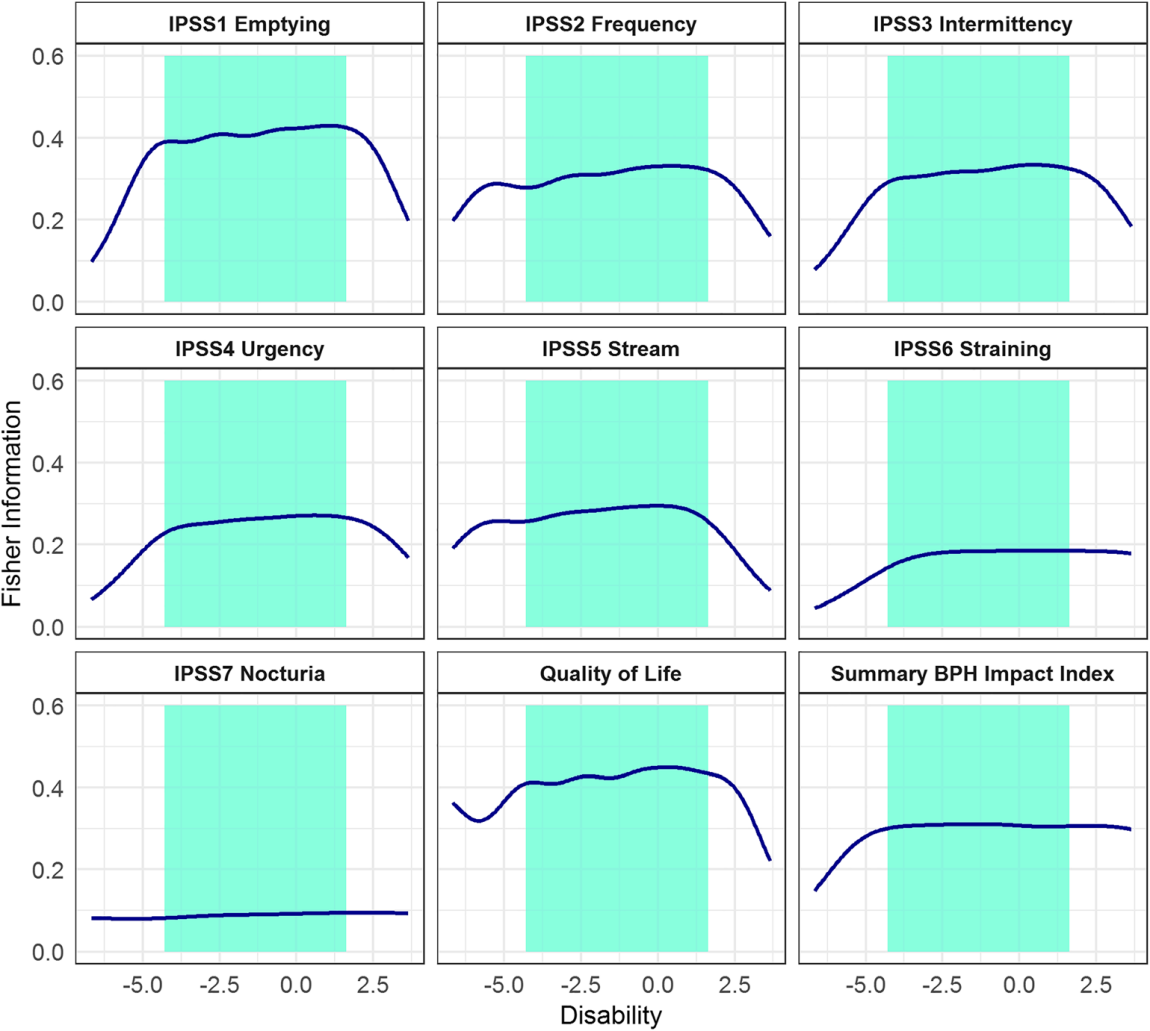
Fisher Information Content of each International Prostate Symptom Score item, the Quality of life score, and the summary Benign Prostatic Hyperplasia (BPH) Impact Index across the estimated disability in the unidimensional integrated item response theory model. Shaded areas indicate the disability range for 95% of the study population

As shown in Fig. 3 and Table II, Fisher Information in the unidimensional integrated BPH-LUTS IRT model ranged from 3.7% for the IPSS *Nocturia* item to 17% for the QoL item. The pooled information content of all IPSS items represented 70.6% of the total information, the pooled information of IPSS voiding items represented 44.7% of the total information, and the pooled information of IPSS storage items represented 25.8% of the total information (Table II).

**Table II:** Fisher Information Content Ranking in the Unidimensional Integrated Item Response Theory Model

Item	Item subscore category	% of total Fisher information	Cumulative % total
Quality of Life score	–	17	17
IPSS1	Voiding	15.4	32.4
IPSS2	Storage	12.4	44.8
BII_summary_	–	12.4	57.2
IPSS3	Voiding	11.9	69.1
IPSS5	Voiding	10.7	79.8
IPSS4	Storage	9.7	89.5
IPSS6	Voiding	6.8	96.3
IPSS7	Storage	3.7	100

*IPSS*, International Prostate Symptom Score; *BII*_*summary*_, Benign Prostatic Hyperplasia Impact Index sum of scores; *IPSS1*, incomplete emptying; *IPSS2*, frequency; *IPSS3*, intermittency; *IPSS4*, urgency; *IPSS5*, weak stream; *IPSS6*, straining; *IPSS7*, nocturia

**Fig. 4:**
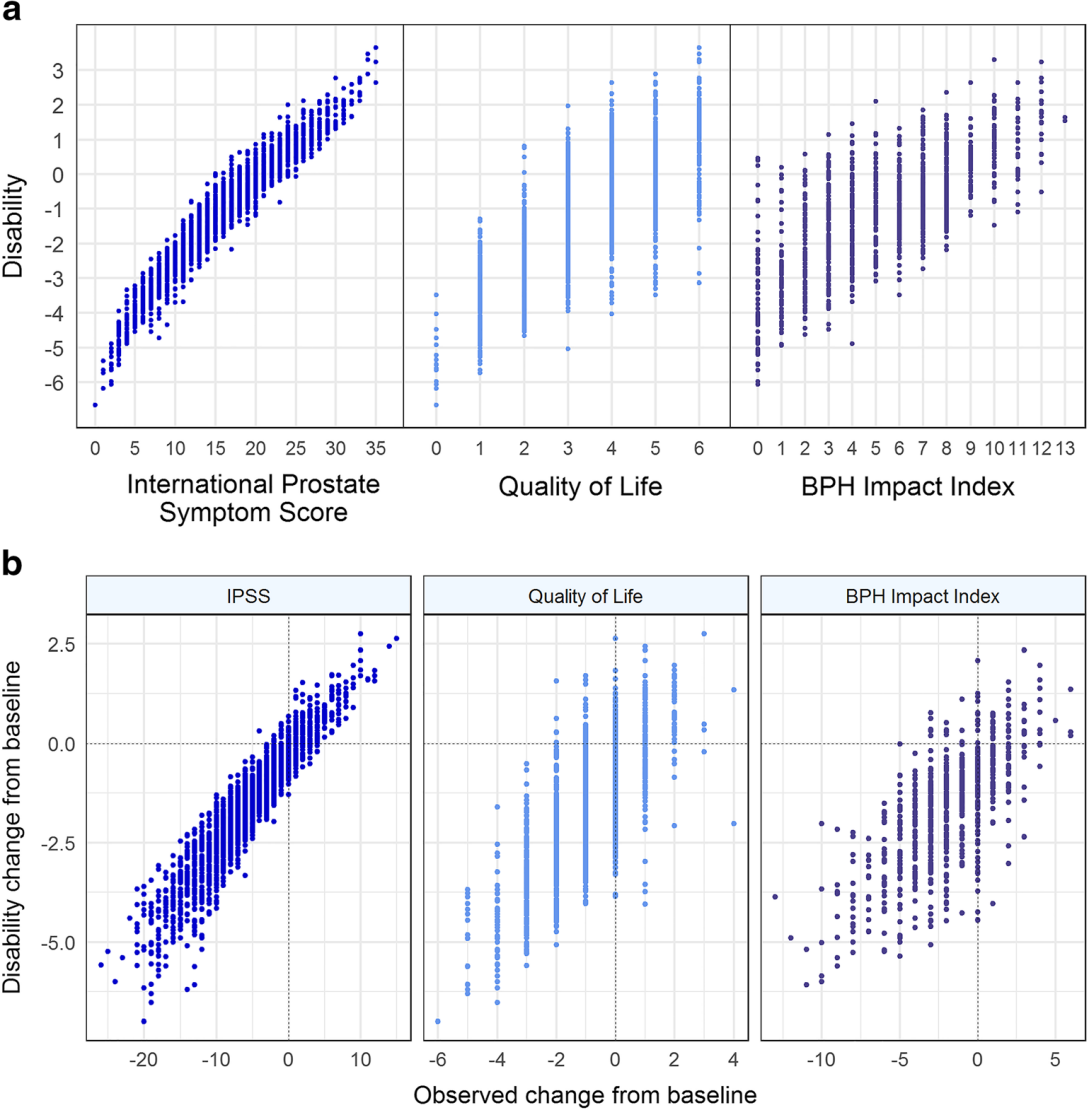
**a** Disability estimated from the unidimensional integrated item response theory model *vs*. the observed total International Prostate Symptom Score (IPSS), the observed Quality of Life (QoL) score, and the observed summary Benign Prostatic Hyperplasia (BPH) Impact Index, respectively, **b** Change from baseline in disability estimates from the unidimensional integrated item response theory model *vs*. observed change from baseline in total IPSS, observed QoL score, and observed summary BPH Impact Index, respectively, in 403 patients over the 6-month trial period

Figure 4a illustrates the relationship between patients’ estimated latent disability in the unidimensional integrated IRT model and their observed total IPSS, observed QoL score, and observed BII_summary_, respectively. High level of agreement was observed between latent disability and total IPSS, QoL, and BII_summary_ (Pearson correlation coefficients of 0.96, 0.77, and 0.71, respectively), indicating that the unidimensional IRT model’s estimate of underlying BPH-LUTS is in line with the observed score from each BPH-LUTS measure. Comparison of the change from baseline in latent disability and the observed change from baseline in each scale is shown in Fig. 4b. Based on the vast majority of the illustrated data, a given patient with observed decreases of at least three, one, and one in total IPSS, QoL, and summary BII, respectively, is expected to have a decrease in latent BPH-LUTS disability. Further specification of the proportion of patients with decreased latent disability at each observed score change is presented in the Supplemental Material.

An exponential model with a drift component described the longitudinal placebo effect and an offset effect described the degarelix treatment effect, similar to the previous longitudinal IRT model considering only IPSS responses (21):

$${\displaystyle \begin{array}{c}\mathrm{Placebo}=P\max \left(1-{e}^{-\frac{\ln (2)}{T\mathrm{prog}}\ast \mathrm{Time}}\right)+\mathrm{Drift}\ast \mathrm{Time}\\ {}\mathrm{Disability}=\mathrm{Baseline}+\mathrm{Placebo}+\mathrm{Drug}\\ {}\mathrm{Drug}=0\kern0.75em \mathrm{if}\ \mathrm{dose}=0\kern0.75em \mathrm{and}\kern0.75em \mathrm{Drug}=\theta \kern0.5em \mathrm{if}\ \mathrm{dose}>0\end{array}}$$

with Baseline being the baseline disability, *P*max the maximal placebo effect, *T*prog the half-life to reach *P*max, Drift the relapse/continued remission parameter, and Drug the offset drug effect of degarelix estimated as a fixed effect (θ). Between-subject variability (BSV) was implemented for Baseline, *P*max, and Drift assuming a normal distribution while BSV was implemented for *T*prog assuming a lognormal distribution. In agreement with the previous finding in the IPSS IRT model (21), no dose-response or exposure-response relationship was observed and such models (slope and Emax) were not found to be significantly better than the effect of degarelix modeled as independent of dose or exposure. Estimation of the drug effect yielded a drop in objective function of 25.0 compared with the reduced model where the fixed effect parameter of degarelix treatment was fixed to zero. Covariate relationships were investigated for the Baseline, *P*max, and Drug parameters. Following backwards elimination, post-void residual volume on Baseline disability was found to be the only significant covariate (*p* < 0.001). The final longitudinal model parameter estimates in the unidimensional BPH-LUTS IRT model are presented in Table III along with their relative standard errors. Categorical VPCs for each item in the unidimensional integrated IRT model are shown in the Supplemental Material, showing adequate model fit for all nine items in all four CS36 treatment arms.

**Table III:** Longitudinal Parameter Estimates for the Unidimensional Item Response Theory (IRT) Model

Parameter	Longitudinal unidimensional integrated IRT model	
	Value	Relative standard error
Baseline	− 0.0993	57.1
*P*max (maximal placebo response)	− 1.22	9.2
*T*prog (placebo half-life)	16.2	17.5
Drug effect	− 0.565	19.3
Covariates		
Post-void residual volume on baseline	0.00327	24.9
Interindividual variability (IIV)		
IIV Baseline	104.4%	5.5
IIV *P*max	134.9%	13.9
IIV Drift	0.9%	9
IIV *T*prog	51.7%	12.6
IIV Baseline-*P*max correlation	15.5%	46.3
IIV *P*max-Drift correlation	45.4%	36.5

### Multidimensional Item Response Theory Modeling

Results of factor analyses with one and two dimensions, respectively, are shown in Table IV. In the bi-dimensional factor analysis, the IPSS storage items and the QoL score were mainly reflected by one dimension while IPSS voiding items were mainly reflected by the other dimension. Moreover, the BII_summary_ item was found to be reflected by both factors to an almost equal extent. Hence, a bidimensional integrated IRT model was developed, where responses to IPSS voiding items were driven by a “voiding” disability, responses to IPSS storage items and the QoL score were driven by a “storage” disability, and a compensatory graded response model allowed for responses of BII_summary_ to be driven by both voiding and storage disability. The ICC parameter estimates in the bi-dimensional integrated IRT model are shown in Table I. Factor analysis using three dimensions showed similar factor loadings to the two-dimensional factor analysis, except for the *Nocturia* item being mainly reflected by the third dimension. As at least three items are needed per latent variable to preserve identification, a three-dimensional IRT model was not pursued.

**Table IV:** Factor Loadings Obtained from Confirmatory Factor Analysis (CFA) of the Nine Items Using One and Two Dimensions, Respectively

Item	Factor loadings using 1 factor	Factor loadings using 2 factors	
		Factor 1	Factor 2
IPSS1	0.756	*− 0.605*	0.462
IPSS2	0.702	− 0.228	*0.763*
IPSS3	0.709	*− 0.672*	0.349
IPSS4	0.662	− 0.310	*0.613*
IPSS5	0.683	*− 0.612*	0.367
IPSS6	0.600	*− 0.806*	0.113
IPSS7	0.459	− 0.108	*0.537*
Quality of Life (QoL)	0.755	− 0.313	*0.752*
Summary BPH Impact Index (BII_summary_)	0.686	*− 0.516*	0.462

In the CFA with two factors, numbers in italic emphasize the largest factor loading value (covariance between each item and factor). Higher factor loadings indicate closer association w*ith* the factor

*IPSS1*, incomplete emptying; *IPSS2*, frequency; *IPSS3*, intermittency; *IPSS4*, urgency; *IPSS5*, weak stream; *IPSS6*, straining; *IPSS7*, nocturia

Similar to the IPSS IRT model (21), a Weibull model was used to describe the longitudinal placebo effect of the underlying disability on each scale:

$$\mathrm{Placebo}=P\max \left(1-{e}^{-{\left(\frac{\ln (2)}{T\mathrm{prog}}\ast \mathrm{Time}\right)}^{\mathrm{WEI}}}\right)+\mathrm{Drift}\ast \mathrm{Time}$$

where WEI is the Weibull exponent. The drug effect model consisted of separate offset effects on each latent variable scale:

$${\displaystyle \begin{array}{c}{\mathrm{Disability}}_{\mathrm{Voiding}}={\mathrm{Baseline}}_{\mathrm{Voiding}}+{\mathrm{Placebo}}_{\mathrm{Voiding}}+{\mathrm{Drug}}_{\mathrm{Voiding}.}\\ {}{\mathrm{Disability}}_{\mathrm{Storage}}={\mathrm{Baseline}}_{\mathrm{Storage}}+{\mathrm{Placebo}}_{\mathrm{Storage}}+{\mathrm{Drug}}_{\mathrm{Storage}}\end{array}}$$

The longitudinal bidimensional integrated model minimized successfully and its parameter estimates are presented in Table V. Due to model instability, it was not possible to obtain parameter uncertainty through the covariance step or perform covariate analysis. With the bidimensional model, a drop in AIC of 2862.2 was observed compared with the unidimensional integrated BPH-LUTS model. Categorical VPCs for each item in the bidimensional integrated IRT model with a compensatory graded response model for the BII_summary_ item are presented in the Supplemental Material.

**Table V:** Parameter Estimates of the Longitudinal Bidimensional Integrated Item Response Theory Model

Parameter	Value
Baseline_V_ (voiding scale)	− 0.0321
Baseline_S_ (storage scale)	− 0.0347
*P*max_V_ (maximal placebo response voiding scale)	− 0.939
*P*max_S_ (maximal placebo response storage scale)	− 1.36
*T*prog_V_ (placebo half-life voiding scale)	12.3
*T*prog_S_ (placebo half-life storage scale)	14
Weibull shape parameter (common for both scales)	1.6
Drug effect voiding scale	− 0.369
Drug effect storage scale	− 0.634
Interindividual variability (IIV)	
IIV Baseline_v_ (voiding scale)	98.6%
IIV Baseline_S_ (storage scale)	128.1%
IIV Baseline_v_-Baseline_S_ correlation	28.9%
IIV *P*max (common for both scales)	114.9%
IIV *T*prog (common for both scales)	60.8%
IIV Drift (common for both scales)	0.6%
IIV *P*max-Drift correlation	33.2%

Interindividual variability was assumed normally distributed for the Baseline, *P*max, and Drift parameters and lognormally distributed for the *T*prog parameter. No relative standard errors were computed due to model stability issues

### Power Determination

**Fig. 5:**
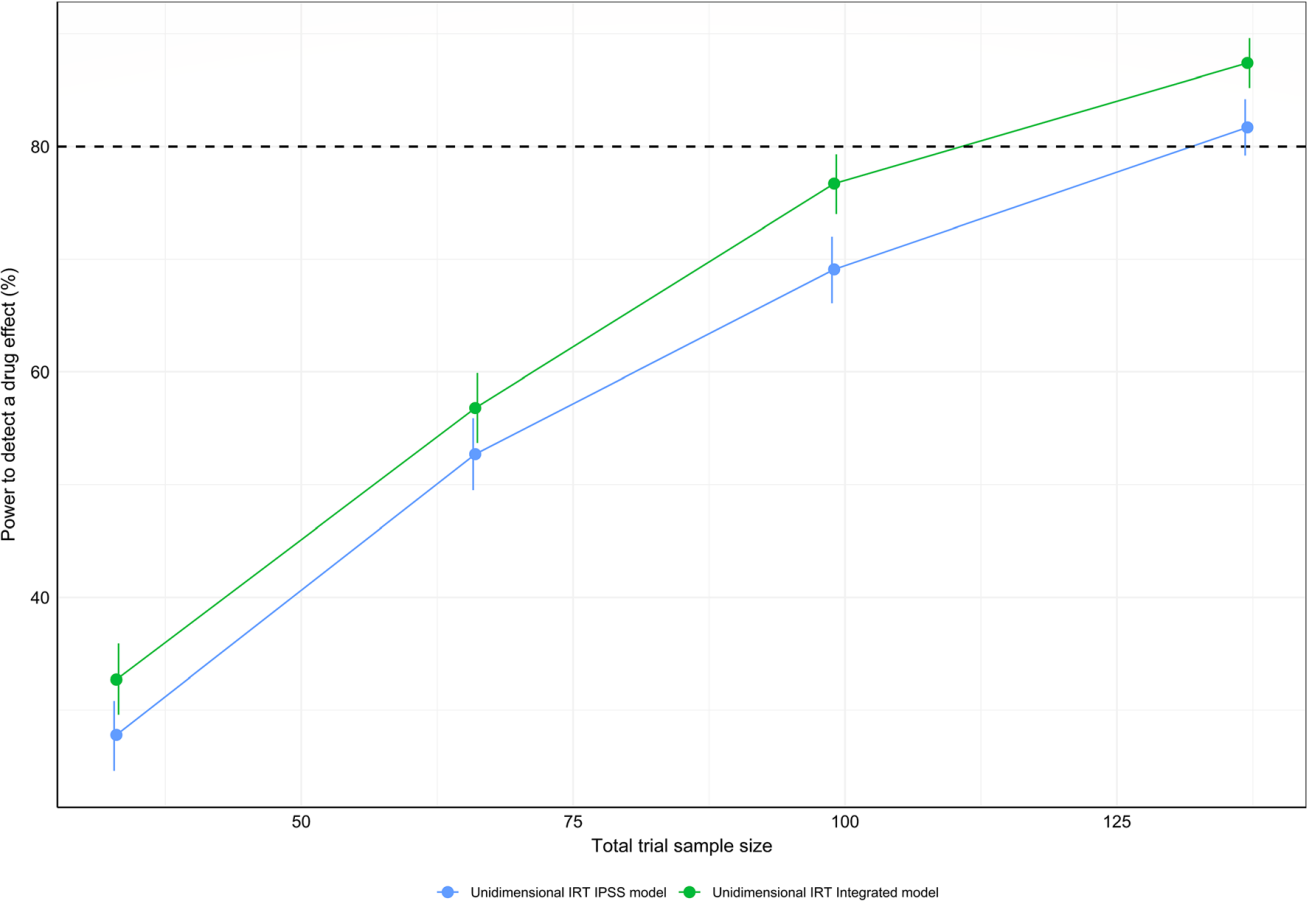
Power curves for the unidimensional integrated and the unidimensional International Prostate Symptom Score (IPSS) item response theory pharmacometric models, respectively, using a stochastic simulation and estimation procedure. One thousand simulated data sets from the integrated unidimensional item response theory model at sample sizes of 33, 66, 99, and 137 patients were used for model estimation with the respective full (with a drug effect parameter) and reduced (without a drug effect parameter) models. Vertical lines indicate the 95% confidence interval for the calculated power estimates. A decrease of 3.84 was used to establish significant improvement in objective function between the full and reduced models of the respective approaches

Figure 5 shows the power of the integrated and IPSS IRT models, respectively. Compared with the unidimensional IRT model considering only IPSS data, the integrated unidimensional IRT model displayed a sample size reduction of 16% to detect a drug effect at 80% power (*N*_IRT-IPSS-Unidimensional_ = 132 *vs*. *N*_IRT-Integrated-Unidimensional_ = 111, well below the actual trial size of 403 patients). At each sample size, the type I error rate was found to be similar in both models (Supplemental Material), and hence, no type-I error adjustment to the power estimates was performed.

## DISCUSSION

The current paper presents models integrating multiple BPH-LUTS scales using IRT. To our knowledge, this is the first model integrating several endpoints within the therapeutic area. We investigated the information content within different BPH-LUTS measures and compared the power to detect a treatment effect of the integrated IRT approach with a previously developed IRT models that considered only IPSS responses. Assessing the effect of drugs on the voiding and storage IPSS subscores is common practice in BPH-LUTS clinical trials although its clinical meaningfulness is not established (13,23,35–37). A previous longitudinal bidimensional IRT model, based on item-level IPSS, aimed to reflect this type of analysis while preserving item-level information (21); in the current work, this model was further extended by including data from the QoL and BII scales. This allowed further characterization of underlying disability and differentiation of the effect of treatment on the “generalized” voiding and storage latent variables, respectively.

In the unidimensional integrated BPH-LUTS IRT model, all scales were modeled assuming a common underlying disability. Unidimensional IRT modeling allows for determination of which item best describes latent disability amongst all items in all BPH-LUTS scales. This overall perspective is lost in a multidimensional IRT setting, since here inference regarding information content can only be made within each scale separately. This may explain why other pharmacometric IRT studies have applied a unidimensional modeling approach to analyze responses from multiple scales (18–20). In the current unidimensional model, the QoL item was found to be the overall most informative, contributing to 17% of the total information content, highlighting the importance of this question for assessing BPH-LUTS. The IPSS *incomplete emptying* item was the second-most informative item, yielding 15.4% of the total Fisher information. This is in line with the previously presented unidimensional IPSS IRT model, where the incomplete emptying item was found to be the most informative (21). Approximately 70% of the total information content was accounted for by the IPSS items, confirming the importance of this scale in characterizing BPH-LUTS and supporting its common use as a primary outcome measure in BPH-LUTS clinical trials. The higher combined information content contribution of IPSS voiding items compared with IPSS storage items is also in line with results from the previous IPSS IRT analysis (21).

The minimal detectable difference (MDD) in observed total IPSS has previously been reported as being at least three points (17) and the current work supports this as decreases in latent disability were strictly observed using this threshold (in accordance with 99.9% of the data). However, as discussed in previous work (21), decreases in latent disability may also be obtained above the MDD, advocating the use of an IRT approach rather than regarding only the summary IPSS to assess patient’s underlying BPH-LUTS. For the QoL score, a decrease of at least one point corresponds to predominantly decreases in IRT-derived latent disability (in 96.8% of patients as shown in the Supplemental Material). This is in line with previous research, where mean QoL reductions ranging 0.5 to 0.8 corresponded to perceived disease improvement in different groups of patients (38). Furthermore, other authors have used a decrease in QoL score of one as this represents a qualitative change on an ordinal scale (39). The current findings may thus have implications for clinical research and the assessment of drug efficacy within BPH-LUTS based on the QoL score. A decrease of at least 0.5 BII points (i.e., 1 point on the observed level) has been reported as the MDD for this scale (17). The current results are in line with this, as this to a large extent corresponds to decreases in latent disability (in 93.6% of patients as shown in the Supplemental Material).

The effect of post-void residual volume (PVR) on baseline latent disability was the only covariate relationship retained in the longitudinal unidimensional integrated IRT model following the stepwise procedure. Weak correlation between symptom severity as expressed by the IPSS and physiologic measures, here amongst PVR, has been reported (40). However, the current finding suggests that post-void residual volume is indicative of underlying BPH-LUTS severity as assessed by several disease-specific scales, and further research should aim to confirm this finding.

Factor analysis with two dimensions indicated that IPSS storage items and the QoL score were predominantly correlated with the same dimension. This is supported by the correlation between IPSS storage items and the QoL score previously highlighted by other authors (15,41–43). High correlation between the BII_summary_ item and both the storage as well as voiding disability was observed, and a compensatory model was used to describe this finding. Each individual BII item may be separately correlated with either the storage or voiding disability, ultimately leading to the BII_summary_ reflecting this. Very limited research has to date been performed examining the level of correlation between individual IPSS and BII items (44), and these indicate that a combination of IPSS voiding and storage items may correlate with the BII_summary_. The compensatory model used in the current work allows for a high value on either the voiding or storage disability scale to potentially compensate for a low value on the other scale, ultimately resulting in a high probability of a BII_summary_ score. It may be of interest to investigate other within-item multidimensional models, such as the non-compensatory/partially compensatory model (45), where high disability on both scales is needed to obtain a high score probability. Due to its complexity and requirement of a larger number of parameters (separate difficulty parameters on each scale), this type of within-item multidimensionality was not investigated in the current work. Compared with a longitudinal bi-dimensional integrated IRT model solely attributing the BII_summary_ item to the voiding latent variable, the compensatory model yielded a drop in AIC of 54.9 points (data not shown).

Incorporating longitudinal QoL and BII scores along with longitudinal item-level IPSS responses in the pharmacometric IRT framework reduced the sample size by 16% to detect a drug effect at 80% compared with considering only item-level IPSS responses. This finding showcases the benefit of utilizing all available information from disease-specific scales within BPH-LUTS to assess treatment effect in a clinical trial setting, made possible by the IRT approach. Quantification of the increase in power to detect a drug effect when simultaneously modeling all scale endpoints as opposed to only considering the primary endpoint marker has to our knowledge not been presented within other therapeutic areas. It may therefore be of interest to further investigate the power of the integrated IRT approach in therapeutic areas where clinical trials commonly include multiple disease-specific scales to assess the treatment effect. The currently reported relative increase in power to detect a drug effect with the integrated unidimensional IRT model is expected to be similar within the context of bidimensional IRT modeling, considering that the difference in modeled data is the same (IPSS, QoL, and BII *vs*. only IPSS). Although the longitudinal bidimensional integrated IRT model yielded a much better fit in terms of likelihood, its complexity and resulting instability may ultimately favor use of the unidimensional approach, which also described the data adequately. For these reasons, comparison of power between the integrated and IPSS bidimensional model, respectively, was not investigated in the current work. Lastly, the observed total IPSS is the common primary endpoint marker in BPH-LUTS clinical trials while pharmacometric IRT focuses on latent disability as the estimand summary measure (46). Pharmacometric IRT possesses higher power to detect a drug effect compared with the total IPSS approach (21), and hence the latter may not be meaningfully applied when the sample size is determined based on IRT-derived latent disability (using only item-level IPSS or multiple BPH-LUTS scales, respectively).

A limitation of the current study is that item-level BII scores were not available for analysis. The information content of each individual BII item is likely to vary, whereas only considering the summary score, as in the current study, assumes it is the same. Accounting for this variation in information content across BII items within the IRT framework may further increase the characterization of BPH-LUTS as well as the power to detect a drug effect of the integrated IRT model. Incorporating summary-level score data as an item, when item-level data are not available, has been reported previously in integrated IRT modeling (19). Although inferior compared with analyzing item-level BII scores, ignoring the BII_summary_ data will lead to a loss of information, as shown by its Fisher Information content contribution in the unidimensional integrated IRT model (Table II). Further, the BII_summary_ was assessed at three visits (baseline, 3 months post-dose, and 6 months post-dose) while item-level IPSS and the QoL score were measured at eight visits. The lower number of BII_summary_ observations may explain the overall higher uncertainty of BII_summary_ difficulty parameters compared with those estimated for individual IPSS items and the QoL score. Moreover, similar to the longitudinal bidimensional IRT model based on item-level IPSS (21) as well as other multidimensional pharmacometric IRT models (24,25), the complexity of the longitudinal bidimensional integrated IRT model led to instability issues. It was hence not possible to obtain the uncertainty of longitudinal parameters or perform covariate analysis for this model. For the same reason, simultaneous estimation of ICCs and longitudinal parameters was not possible. More advanced and time-consuming techniques such as the bootstrap may be used to obtain parameter precision but was not performed here. The longitudinal bidimensional integrated IRT model minimized succesfully and showed adequate item and summary-level data description as assessed through VPCs; the longitudinal parameters were therefore ultimately deemed trustworthy. Lastly, in the longitudinal unidimensional integrated IRT model, simultaneously estimating the ICCs and longitudinal parameters decreased the objective function value by 16.7 points (data not shown) compared with fixing the ICCs. This was not significant given 63 degrees of freedom (63 ICC parameters) under a *χ*^2^ distribution.

Although validated to assess BPH-LUTS (16,17,47), neither the IPSS, the QoL score, or the BII assesses incontinence, which may be an important and bothersome symptom in patients with BPH-LUTS (14,48). Extending the current models to include such information using, e.g., the Incontinence Severity Index (49), the Epidemiology of LUTS questionnaire (50), and/or the International Consultation on Incontinence Modular Questionnaire (51) may further enhance the characterization of BPH-LUTS and its progression as well as further increase the power to detect a drug effect compared with only regarding the IPSS. In this context, it may also be of benefit to investigate the inclusion of generic PROs such as, e.g., the EuroQol-5 Domain scale (52) (EQ-5D) and the Visual Analogue Scale (53,54), potentially while guiding responses from these scales towards BPH-LUTS using a supervised IRT approach (55,56).

## CONCLUSION

IRT modeling was used to integrate data from multiple disease-specific PRO endpoints within BPH-LUTS into a single model. A sample size reduction of 16% to detect a drug effect at 80% power was obtained with the unidimensional integrated IRT model compared with its counterpart IPSS IRT model. This study shows that utilizing the information content across IPSS, QoL, and BII scales in an integrated IRT framework results in a modest but meaningful increase in power to detect a drug effect.

## Electronic Supplementary Material

ESM 1 (PDF 3837 kb)

ESM 2 (PDF 247 kb)

ESM 3 (PDF 439 kb)
