# Supplementary material for: Integrated Item Response Theory Modeling of Multiple Patient-Reported Outcomes Assessing Lower Urinary Tract Symptoms Associated with Benign Prostatic Hyperplasia
Source: AAPS J. 2020 Jul 29;22(5):98. doi: 10.1208/s12248-020-00484-7 (PMC7391402; doi:10.1208/s12248-020-00484-7)
Supplement: Supplementary file 1 — (PDF 3837 kb) [file 12248_2020_484_MOESM1_ESM.pdf]

Supplemental Material

Integrated Item Response Theory Modeling of Multiple Patient Reported Outcomes Assessing Lower Urinary Tract Symptoms associated with Benign Prostatic Hyperplasia

Table of Contents

1. **CS36 data overview** 2
   1.1 Item-level International Prostate Symptom Scores 2
   1.2 Quality of Life scores 3
   1.3 Summary Benign Prostatic Hyperplasia Impact Index 5
2. **Unidimensional item response theory model item characteristic curves model diagnostics** 7
   2.1 International Prostate Symptom Score items 7
   2.2 Quality of Life item 8
   2.3 Summary Benign Prostatic Hyperplasia Impact Index item 8
3. **Latent disability change from baseline vs. observed change from baseline** 9
4. **Longitudinal Unidimensional Integrated Item Response Theory model** 10
   4.1 Item-level visual predictive checks 10
      4.1.1 International Prostate Symptom Score Item 1 10
      4.1.2 International Prostate Symptom Score Item 2 11
      4.1.3 International Prostate Symptom Score Item 3 12
      4.1.4 International Prostate Symptom Score Item 4 13
      4.1.5 International Prostate Symptom Score Item 5 14
      4.1.6 International Prostate Symptom Score Item 6 15
      4.1.7 International Prostate Symptom Score Item 7 16
      4.1.8 Quality of Life score 17
      4.1.9 Benign Prostatic Hyperplasia Impact Index 18
5. **Bidimensional Item response Theory Item parameter estimates** 19
6. **Longitudinal bidimensional integrated item response theory model** 21
   6.1 Item-level visual predictive checks 21
      6.1.1 International Prostate Symptom Score Item 1 21
      6.1.2 International Prostate Symptom Score Item 2 22
      6.1.3 International Prostate Symptom Score Item 3 23
      6.1.4 International Prostate Symptom Score Item 4 24
      6.1.5 International Prostate Symptom Score Item 5 25
      6.1.6 International Prostate Symptom Score Item 6 26
      6.1.7 International Prostate Symptom Score Item 7 27
      6.1.8 Quality of Life score 28
      6.1.9 Benign Prostatic Hyperplasia Impact Index 29
7. **Type I error in Stochastic Simulation and Estimation procedure** 30

1. CS36 data overview

1.1 Item-level International Prostate Symptom Scores

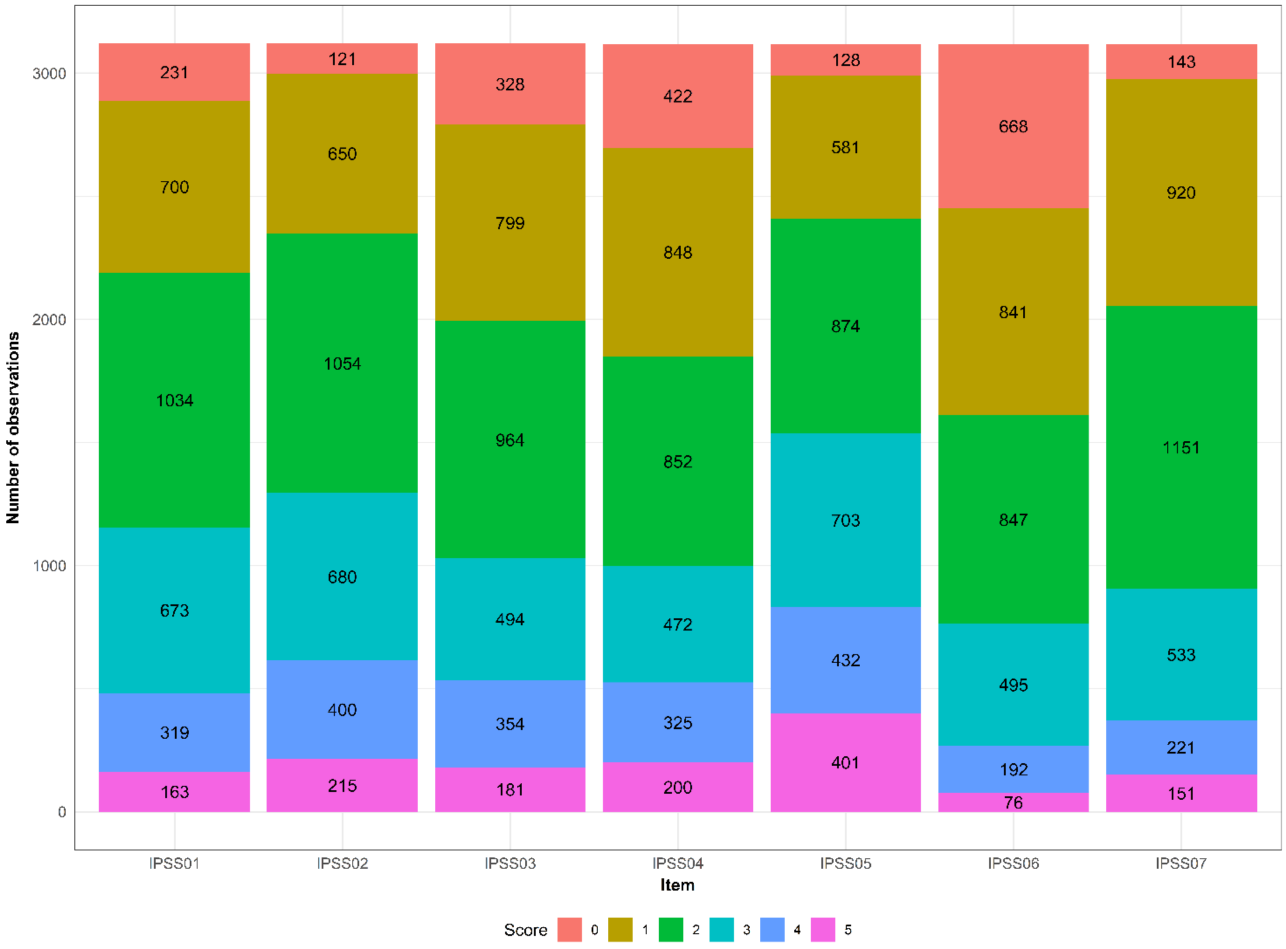

Figure S1.1 – Score distribution in the CS36 trial for each of the seven International Prostate Symptom Score items used in integrated item response theory model building. IPSS01: Item 1 "Incomplete Emptying"; IPSS02: Item 2 "Frequency"; IPSS03: Item 3 "Intermittency"; IPSS04: Item 4 "Urgency"; IPSS05: Item 5 "Weak Stream"; IPSS06: Item 6 "Straining"; IPSS07: Item 7 "Nocturia".

1.2 Quality of Life scores

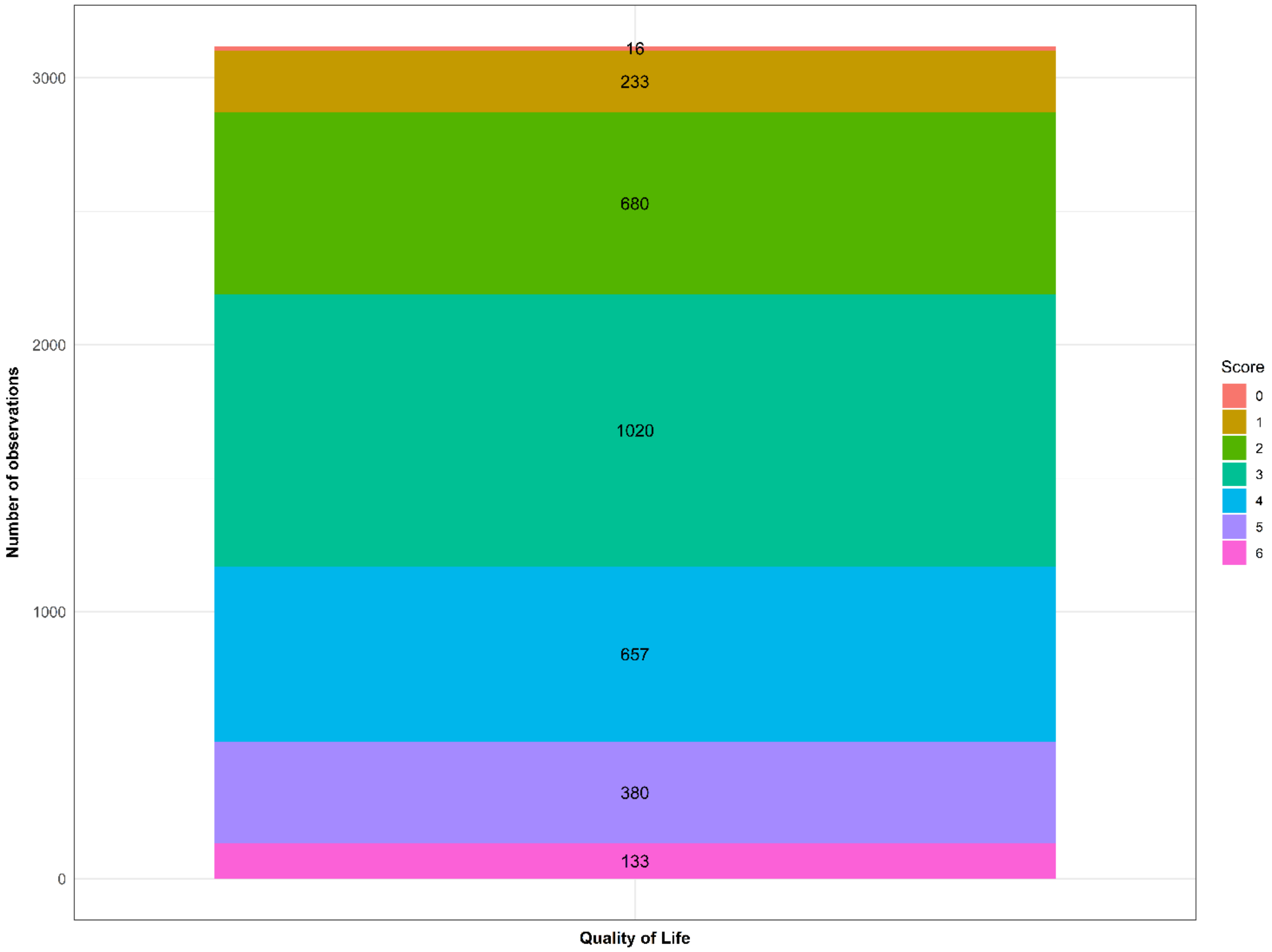

Figure S1.2 – Score distribution in the CS36 trial for the Quality of Life (QoL) Score item used in integrated item response theory model building.

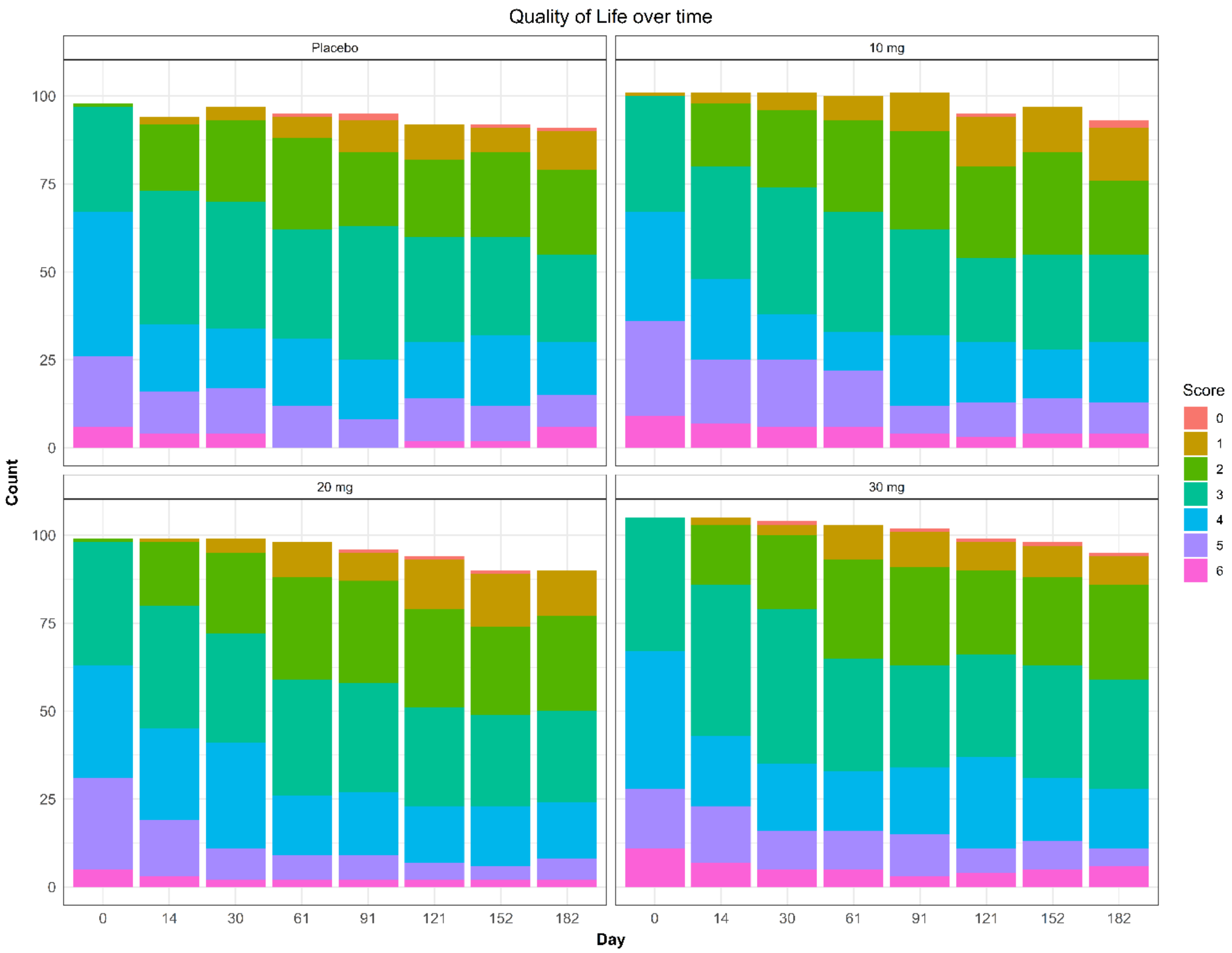

Figure S1.3 – Change in the number of observed Quality of Life (QoL) scores over time in each CS36 clinical trial arm.

1.3 Summary Benign Prostatic Hyperplasia Impact Index

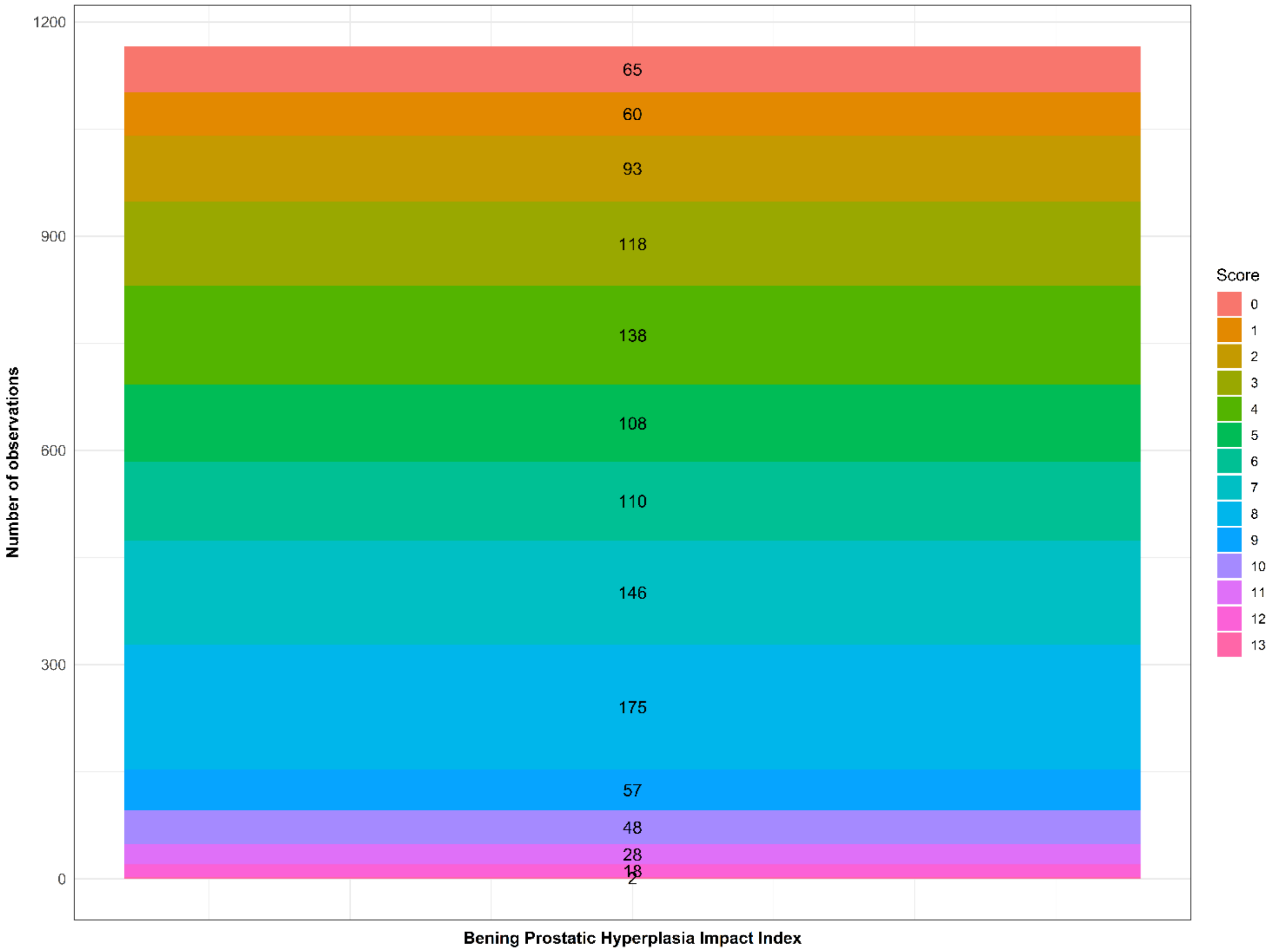

Figure S1.4 – Score distribution in the CS36 trial for the summary Benign Prostatic Hyperplasia Impact Index ($BII_{summary}$) item used in integrated item response theory model building.

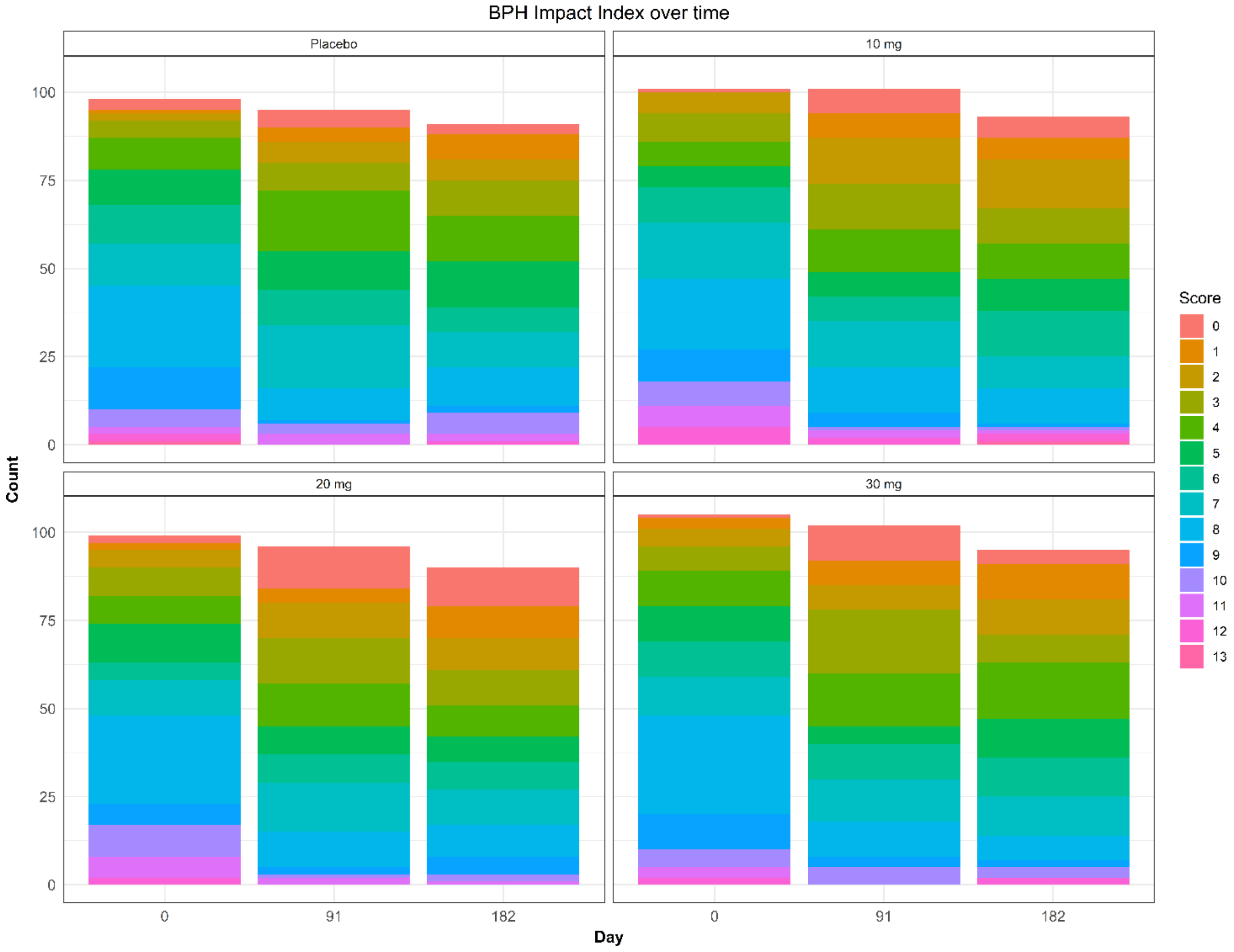

Figure S1.5 – Change in the number of observed summary Benign Prostatic Hyperplasia (BPH) Impact Index ($BII_{summary}$) scores over time in each CS36 clinical trial arm.

2. Unidimensional item response theory model item characteristic curves model diagnostics

2.1 International Prostate Symptom Score items

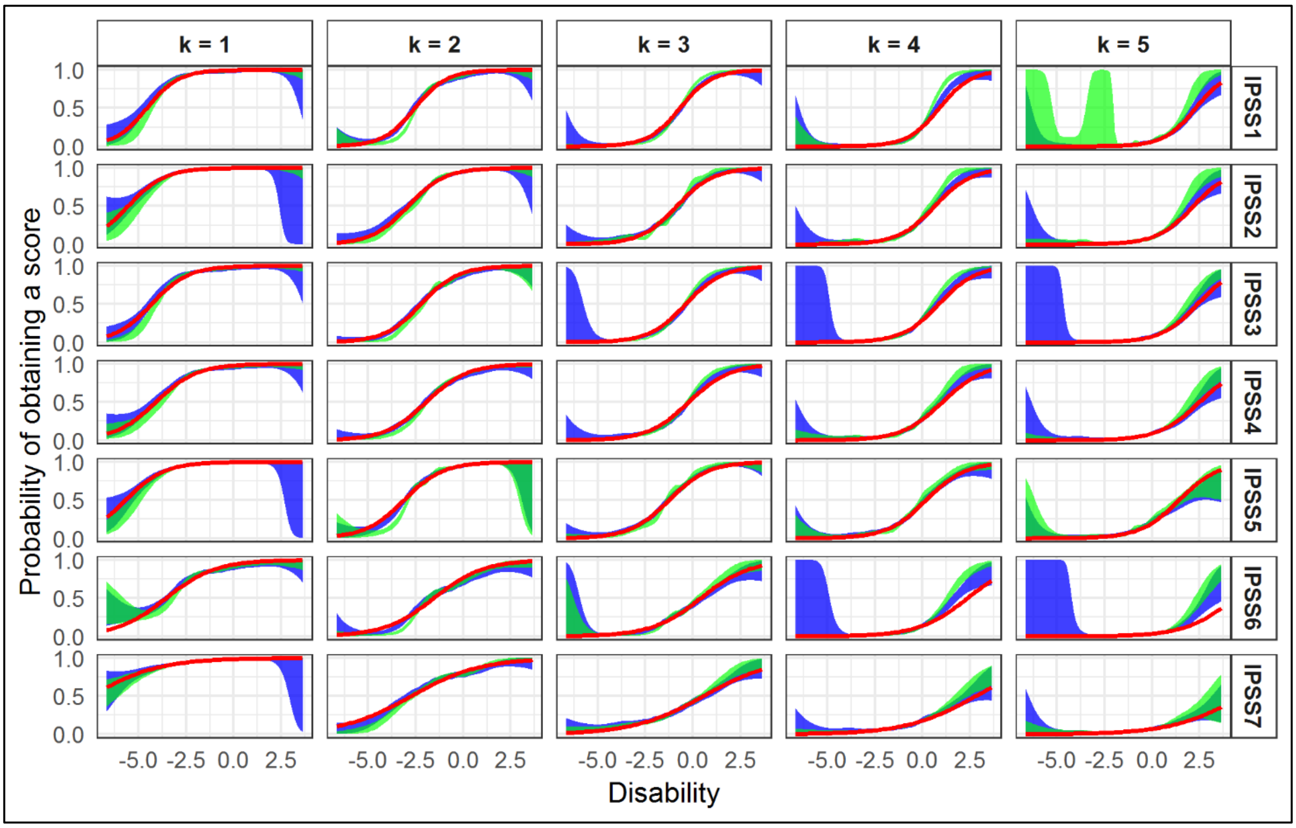

Figure S2.1 - International Prostate Symptom Score (IPSS) item characteristic curve fits in the unidimensional integrated item response theory model for the cumulative probabilities (red lines) along with cross-validated cubic spline generalized additive model (GAM) smooth (green area) and η sampling-based cross-validated cubic spline GAM smooth using 200 samples (blue area). k is the score category (i.e. the dependent variable (DV)). IPSS1: Item 1 “Incomplete Emptying”; IPSS2: Item 2 “Frequency”; IPSS3: Item 3 “Intermittency”; IPSS4: Item 4 “Urgency”; IPSS5: Item 5 “Weak Stream”; IPSS6: Item 6 “Straining”; IPSS7: Item 7 “Nocturia”.

2.2 Quality of Life item

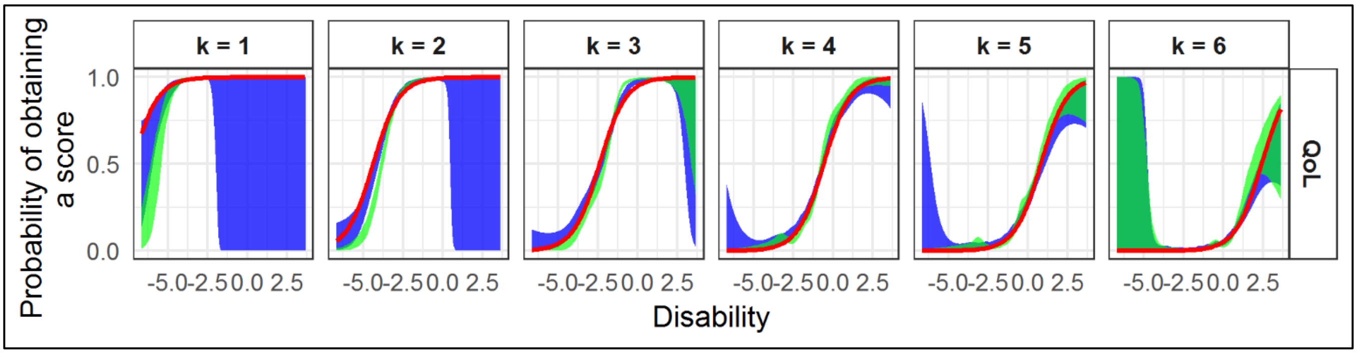

Figure S2.2 – Quality of Life (QoL) score characteristic curve fits in the unidimensional integrated item response theory model for the cumulative probabilities (red lines) along with cross-validated cubic spline generalized additive model (GAM) smooth (green area) and η sampling-based cross-validated cubic spline GAM smooth using 200 samples (blue area). k is the score category (i.e. the dependent variable (DV)).

2.3 Summary Benign Prostatic Hyperplasia Impact Index item

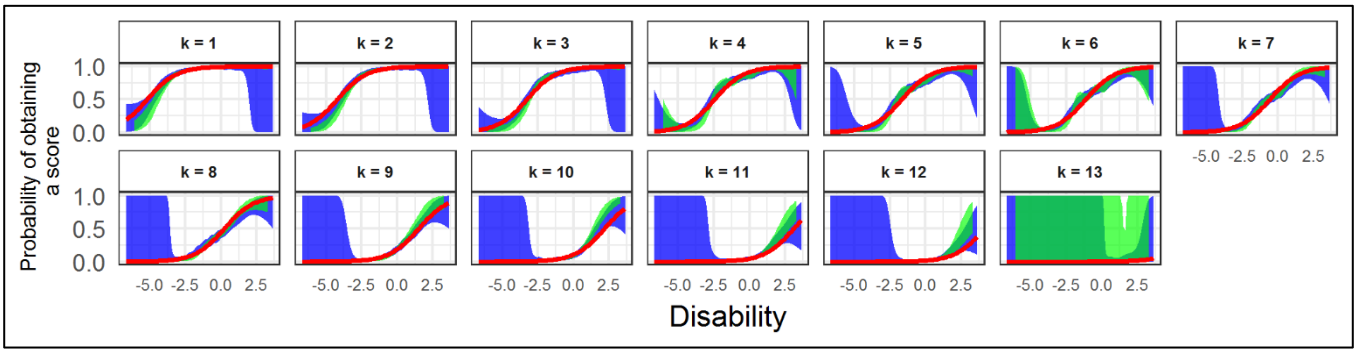

Figure S2.3 – Benign Prostatic Hyperplasia Impact Index score characteristic curve fits in the unidimensional integrated item response theory model for the cumulative probabilities (red lines) along with cross-validated cubic spline generalized additive model (GAM) smooth (green area) and η sampling-based cross-validated cubic spline GAM smooth using 200 samples (blue area). k is the score category (i.e. the dependent variable (DV)).

3. Latent disability change from baseline vs. observed change from baseline

Table S3 – Proportion of measurements with increase in change from baseline disability (>0) in the unidimensional integrated item characteristic curve estimation model at observed changes from baseline in each BPH-LUTS scale in Figure 4b. IPSS: International Prostate Symptom, QoL: Quality of Life, BII: Benign Prostatic Hyperplasia Impact Index.

Change from baseline in observed score	Proportion of increases in change from baseline latent disability	Total number of measurements at observed change from baseline score
IPSS		
0	13%	161
-1	6.5%	201
-2	2.3%	259
-3	0%	259
-4	0.64%	158
QoL		
0	16%	842
-1	3.2%	963
-2	0.7%	468
BII		
0	9.5%	116
-1	6.4%	109
-2	3.5%	115
-3	7.1%	99

4. Longitudinal Unidimensional Integrated Item Response Theory model

4.1 Item-level visual predictive checks

4.1.1 International Prostate Symptom Score Item 1

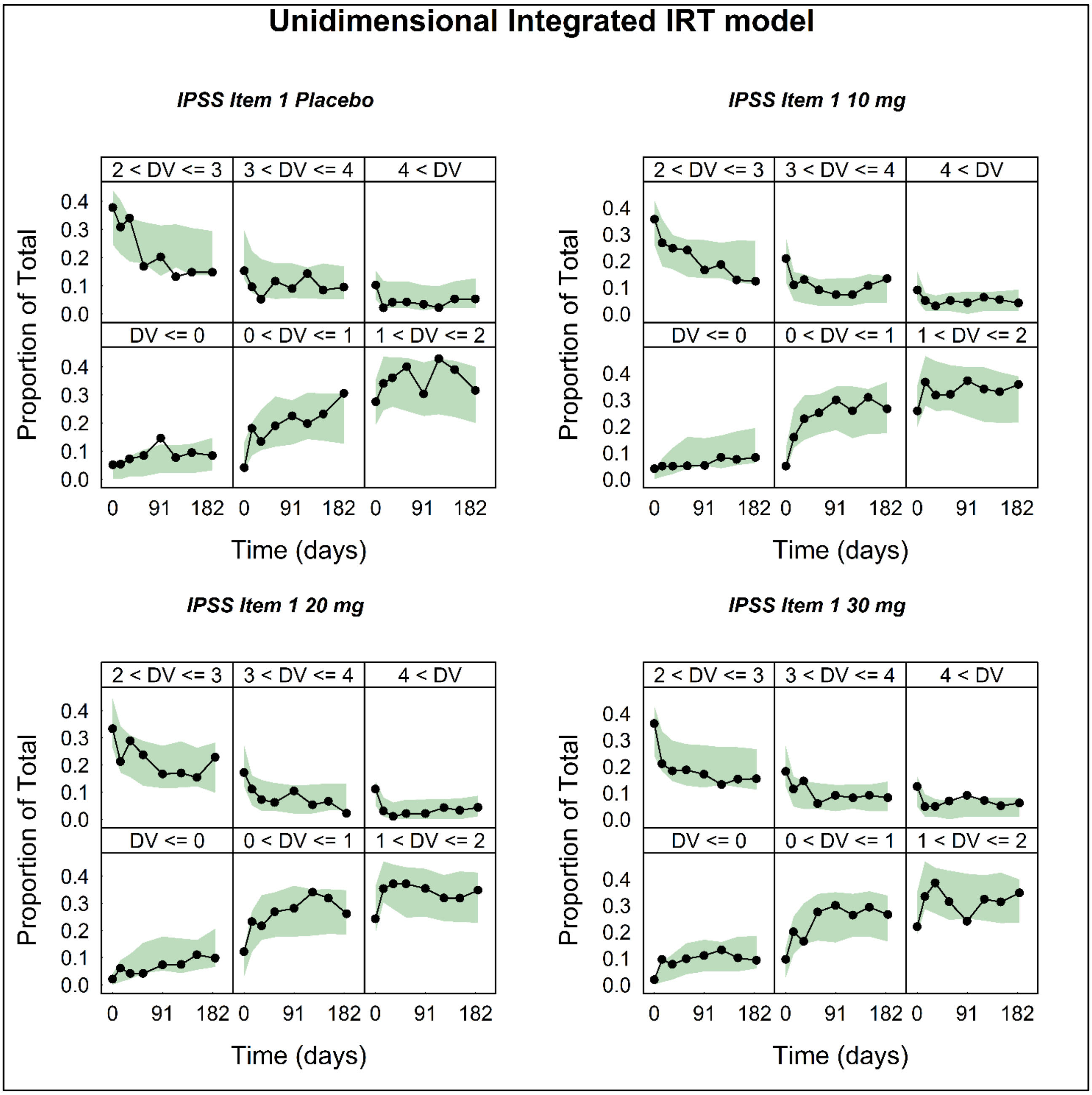

Figure S4.1 –Visual predictive check for the International Prostate Symptom Score (IPSS) item 1 "Incomplete Emptying" in the unidimensional integrated item response theory (IRT) model stratified by treatment arm. The observed frequency of each score over time is shown as points and the shaded areas

indicate the 95% confidence intervals of the frequencies of each score in 200 simulated datasets. DV: Dependent variable, i.e. observed score or score category (k).

4.1.2 International Prostate Symptom Score Item 2

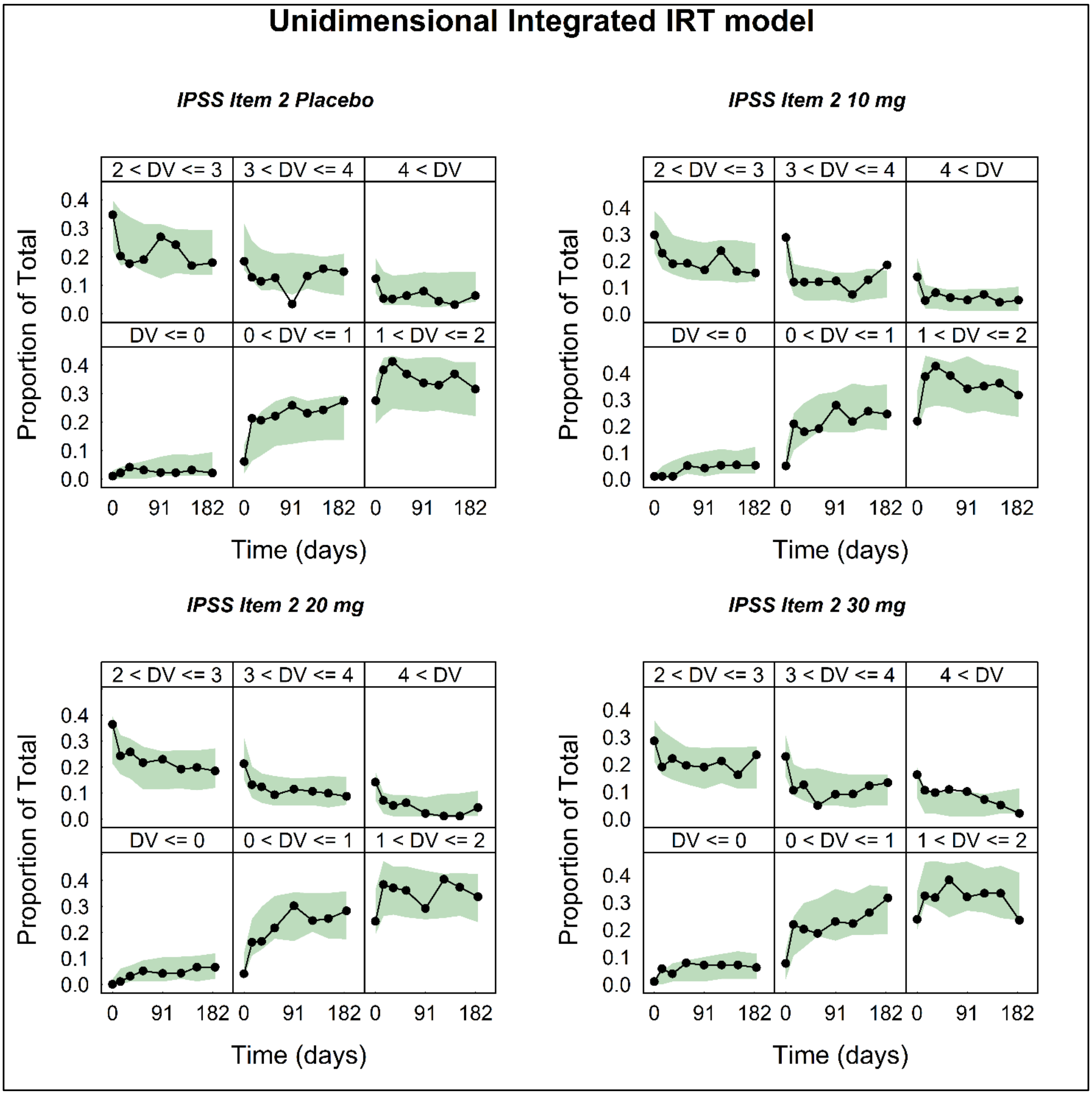

Figure S4.2 –Visual predictive check for the International Prostate Symptom Score (IPSS) item 2 "Frequency" in the unidimensional integrated item response theory (IRT) model stratified by treatment arm. The observed frequency of each score over time is shown as points and the shaded areas indicate the 95% confidence intervals of the frequencies of each score in 200 simulated datasets. DV: Dependent variable, i.e. observed score or score category (k).

4.1.3 International Prostate Symptom Score Item 3

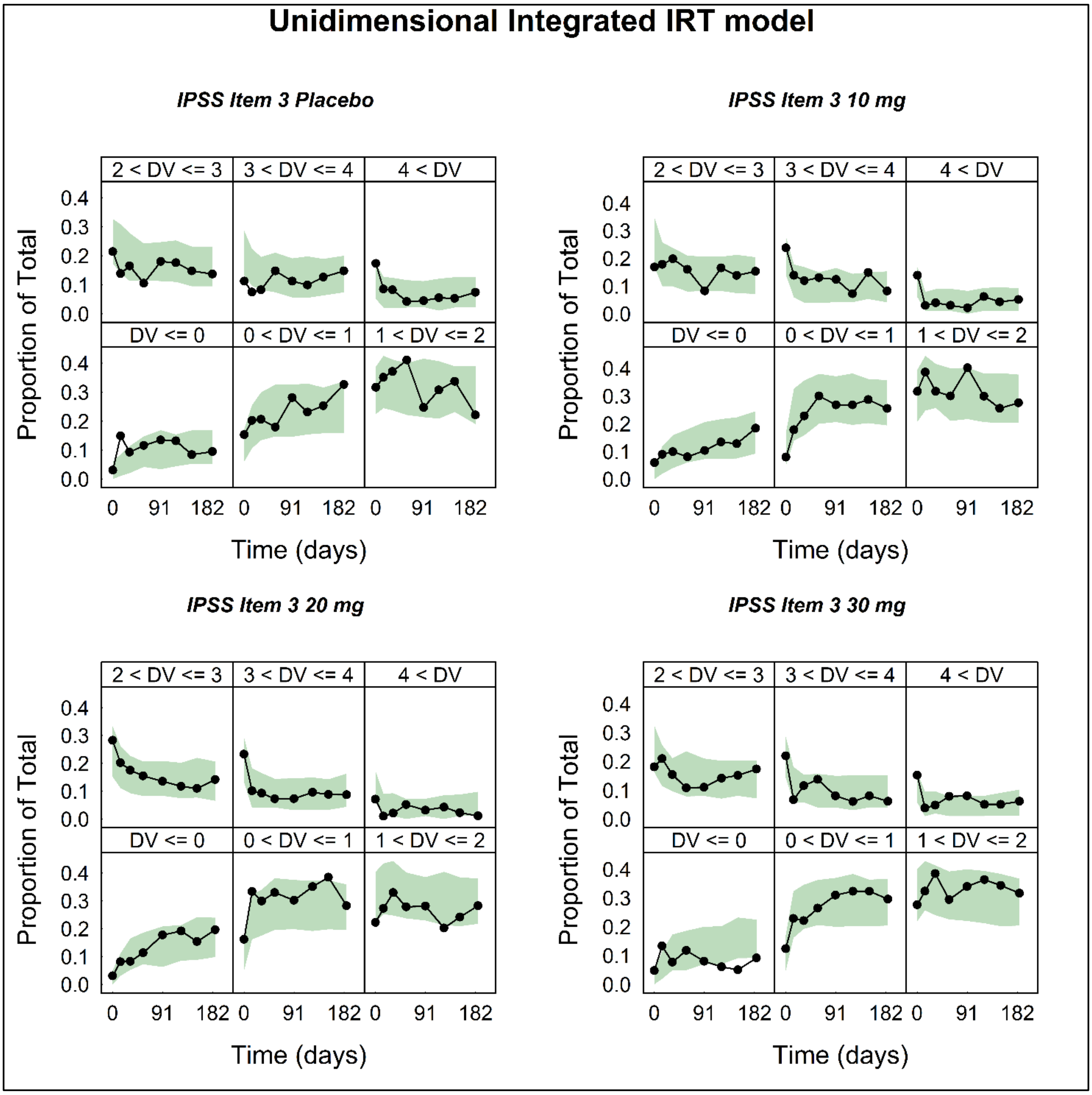

Figure S4.3 –Visual predictive check for the International Prostate Symptom Score (IPSS) item 3 "Intermittency" in the unidimensional integrated item response theory (IRT) model stratified by treatment arm. The observed frequency of each score over time is shown as points and the shaded areas indicate the 95% confidence intervals of the frequencies of each score in 200 simulated datasets. DV: Dependent variable, i.e. observed score or score category (k).

4.1.4 International Prostate Symptom Score Item 4

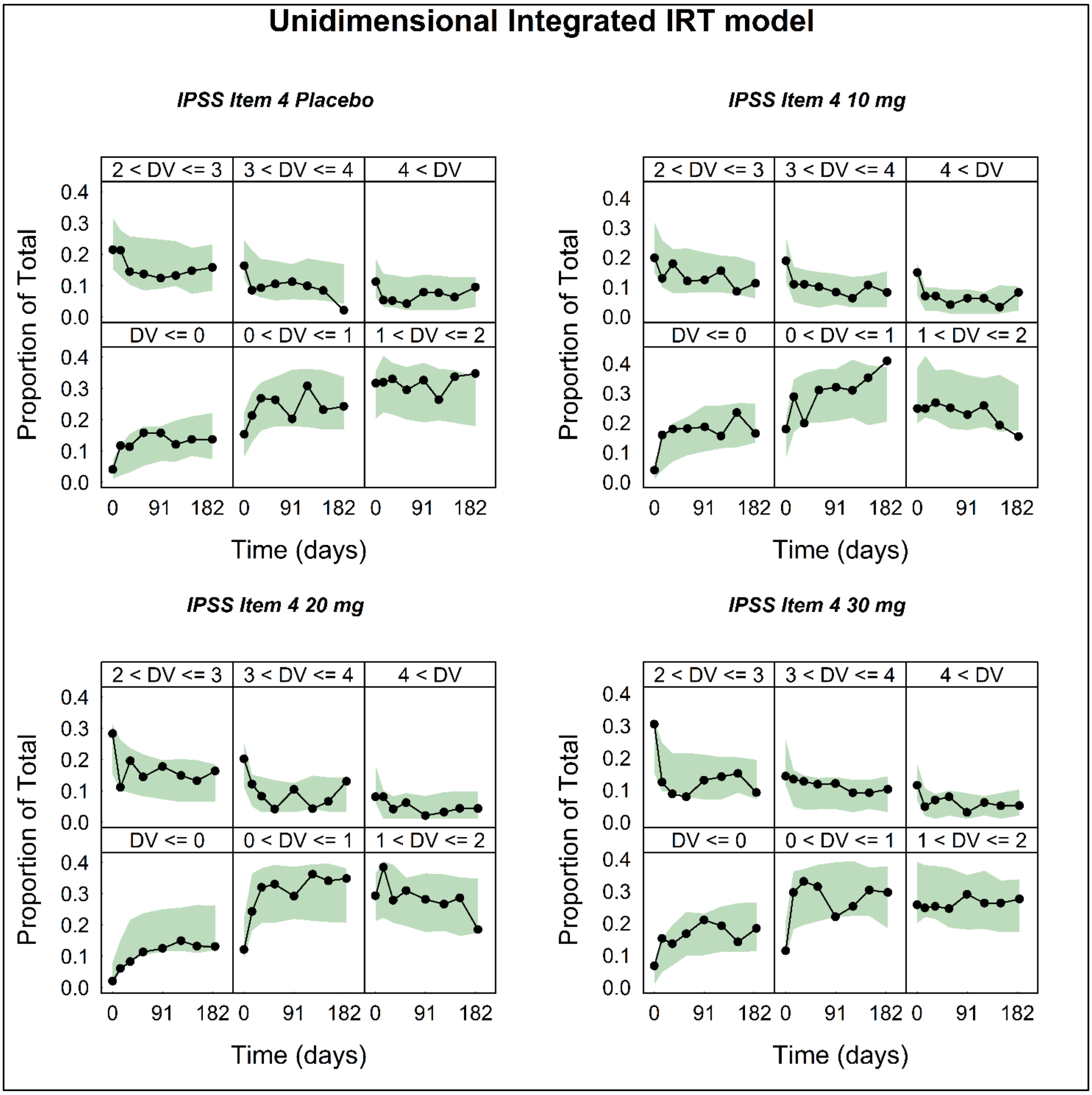

Figure S4.4 –Visual predictive check for the International Prostate Symptom Score (IPSS) item 4 "Urgency" in the unidimensional integrated item response theory (IRT) model stratified by treatment arm. The observed frequency of each score over time is shown as points and the shaded areas indicate the 95% confidence intervals of the frequencies of each score in 200 simulated datasets. DV: Dependent variable, i.e. observed score or score category (k).

4.1.5 International Prostate Symptom Score Item 5

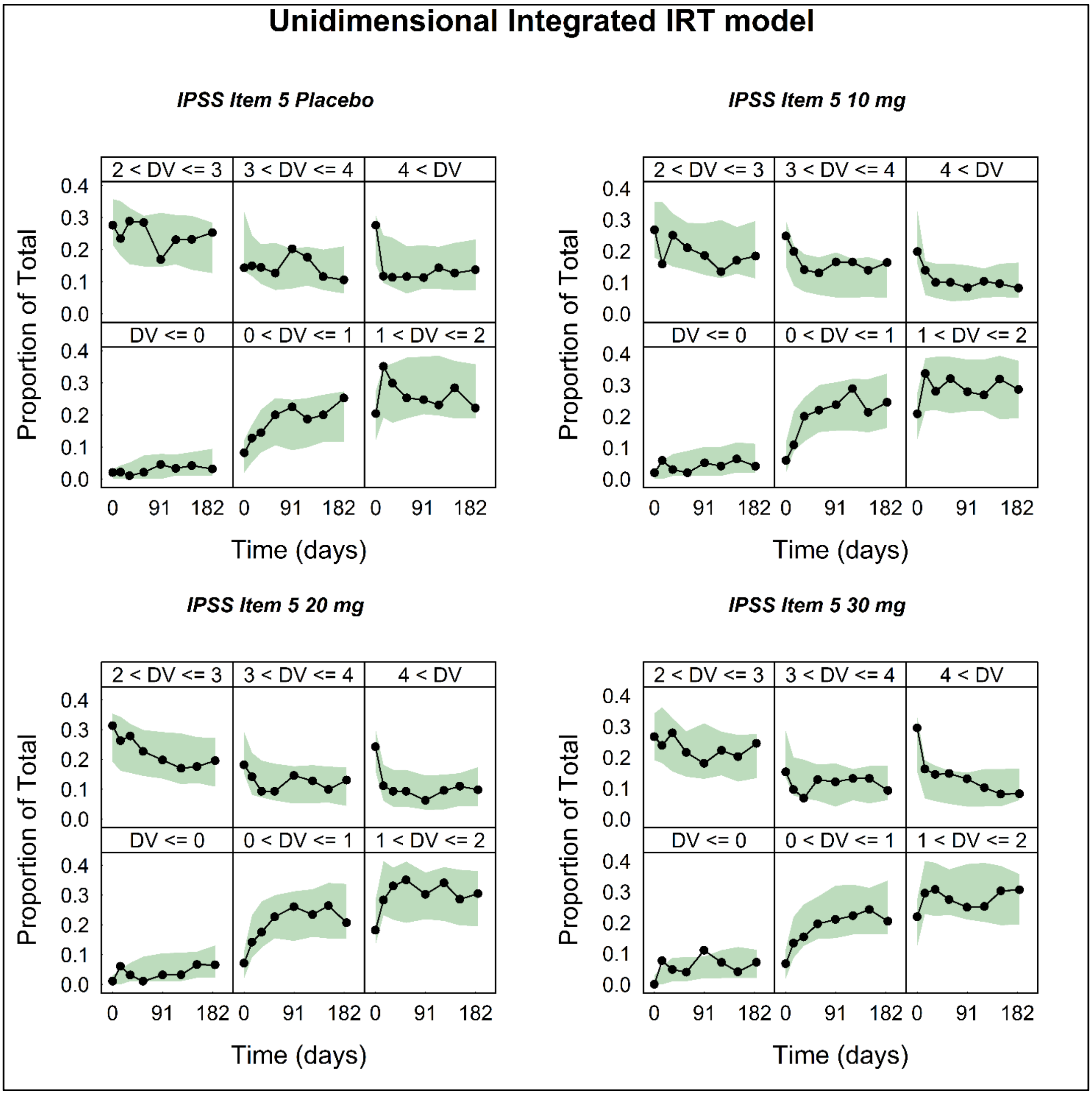

Figure S4.5 –Visual predictive check for the International Prostate Symptom Score (IPSS) item 5 "Weak Stream" in the unidimensional integrated item response theory (IRT) model stratified by treatment arm. The observed frequency of each score over time is shown as points and the shaded areas indicate the 95% confidence intervals of the frequencies of each score in 200 simulated datasets. DV: Dependent variable, i.e. observed score or score category (k).

4.1.6 International Prostate Symptom Score Item 6

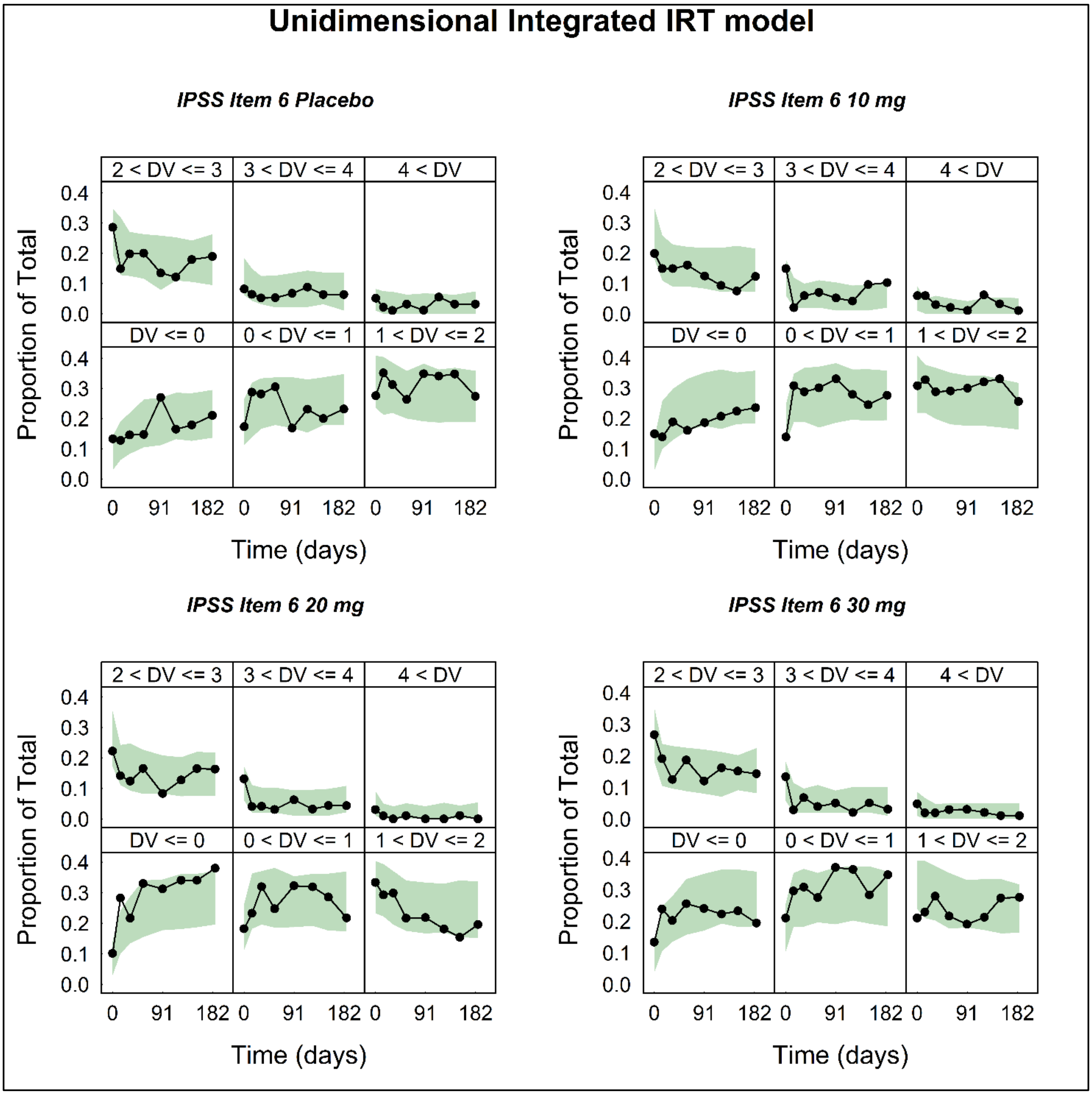

Figure S4.6 –Visual predictive check for the International Prostate Symptom Score (IPSS) item 6 "Straining" in the unidimensional integrated item response theory (IRT) model stratified by treatment arm. The observed frequency of each score over time is shown as points and the shaded areas indicate the 95% confidence intervals of the frequencies of each score in 200 simulated datasets. DV: Dependent variable, i.e. observed score or score category (k).

4.1.7 International Prostate Symptom Score Item 7

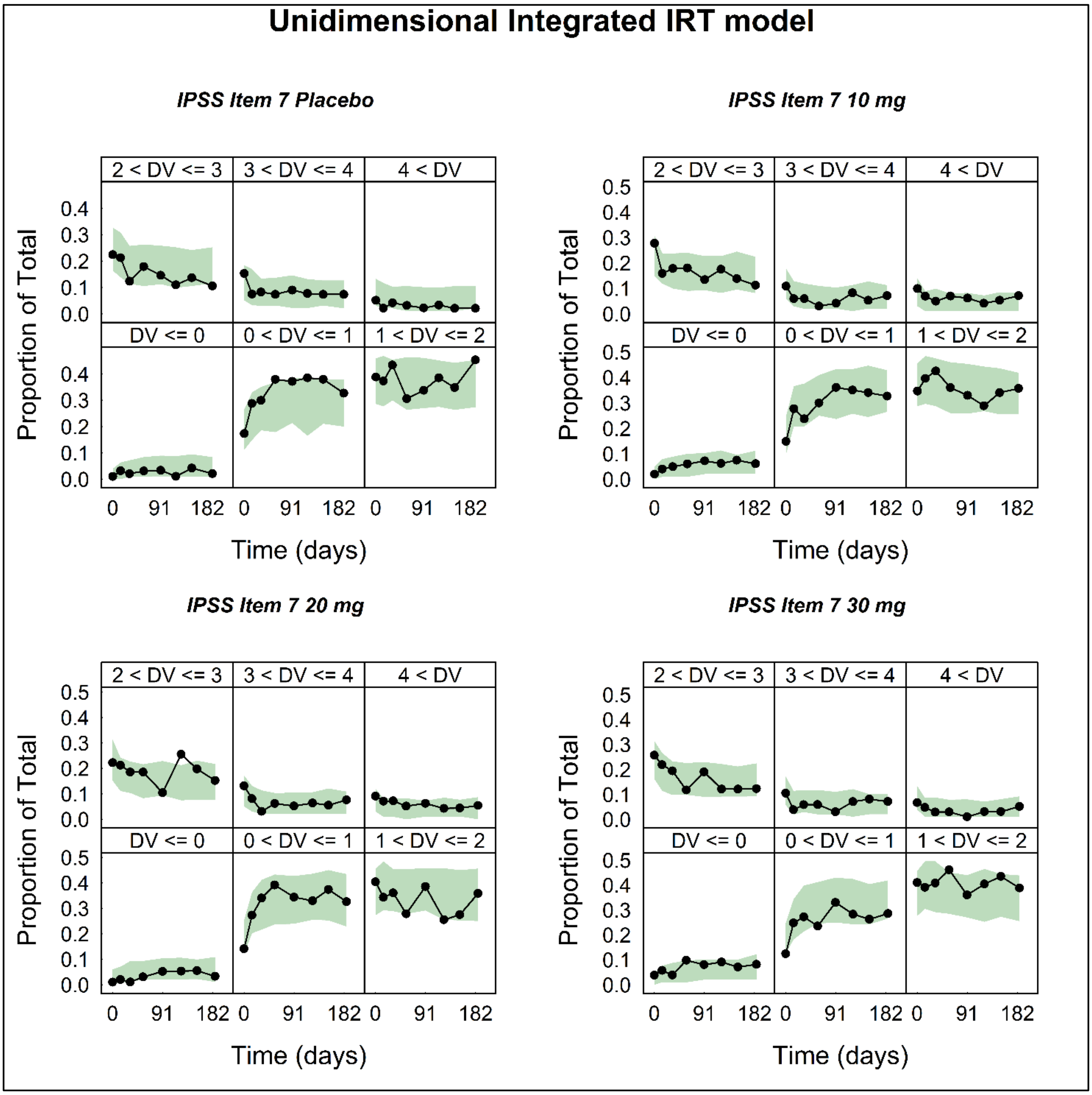

Figure S4.7 –Visual predictive check for the International Prostate Symptom Score (IPSS) item 7 "Nocturia" in the unidimensional integrated item response theory (IRT) model stratified by treatment arm. The observed frequency of each score over time is shown as points and the shaded areas indicate the 95% confidence intervals of the frequencies of each score in 200 simulated datasets. DV: Dependent variable, i.e. observed score or score category (k).

4.1.8 Quality of Life score

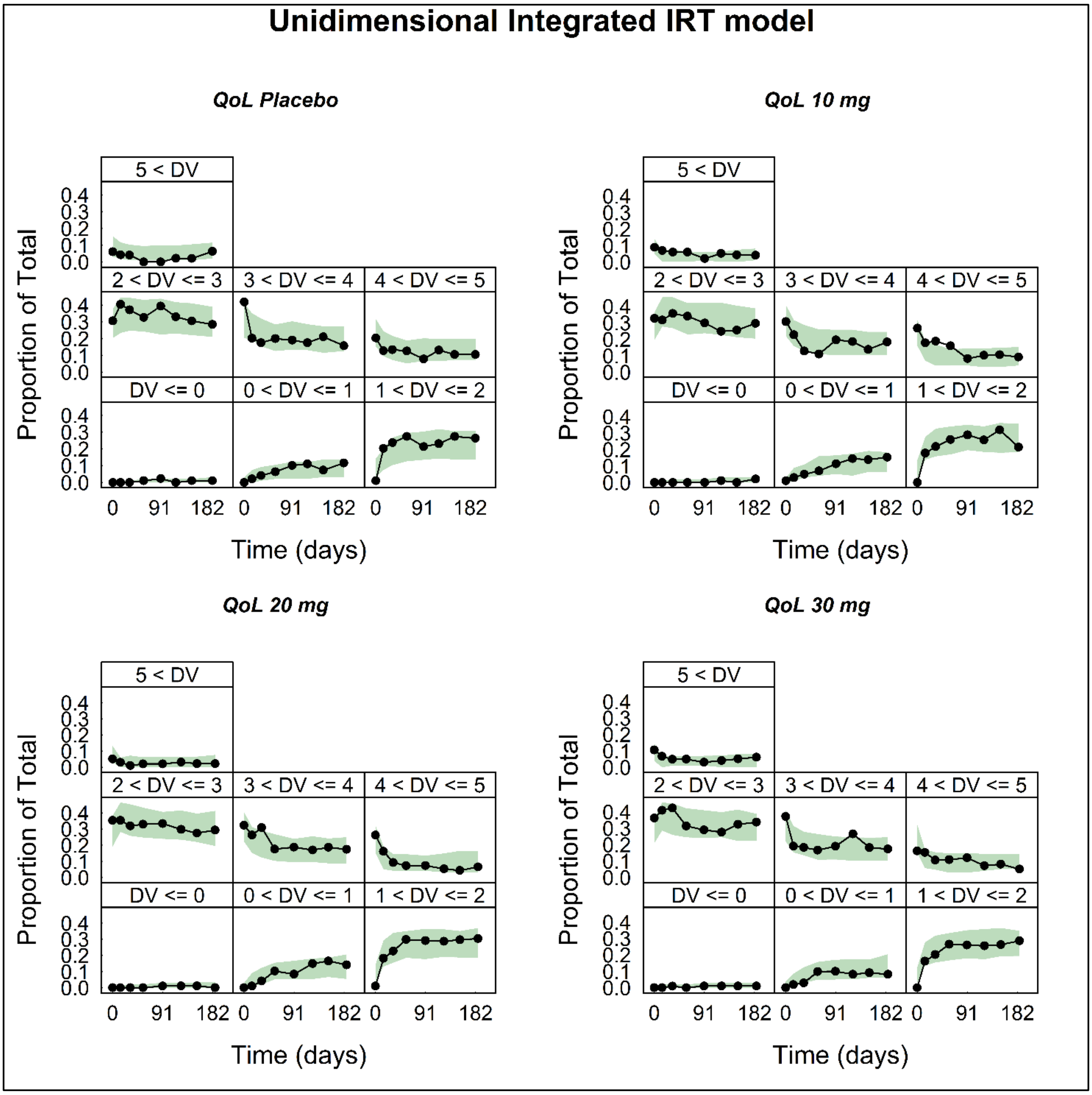

Figure S4.8 –Visual predictive check for the Quality of Life (QoL) Score in the unidimensional integrated item response theory (IRT) model stratified by treatment arm. The observed frequency of each score over time is shown as points and the shaded areas indicate the 95% confidence intervals of the frequencies of each score in 200 simulated datasets. DV: Dependent variable, i.e. observed score or score category (k).

4.1.9 Benign Prostatic Hyperplasia Impact Index

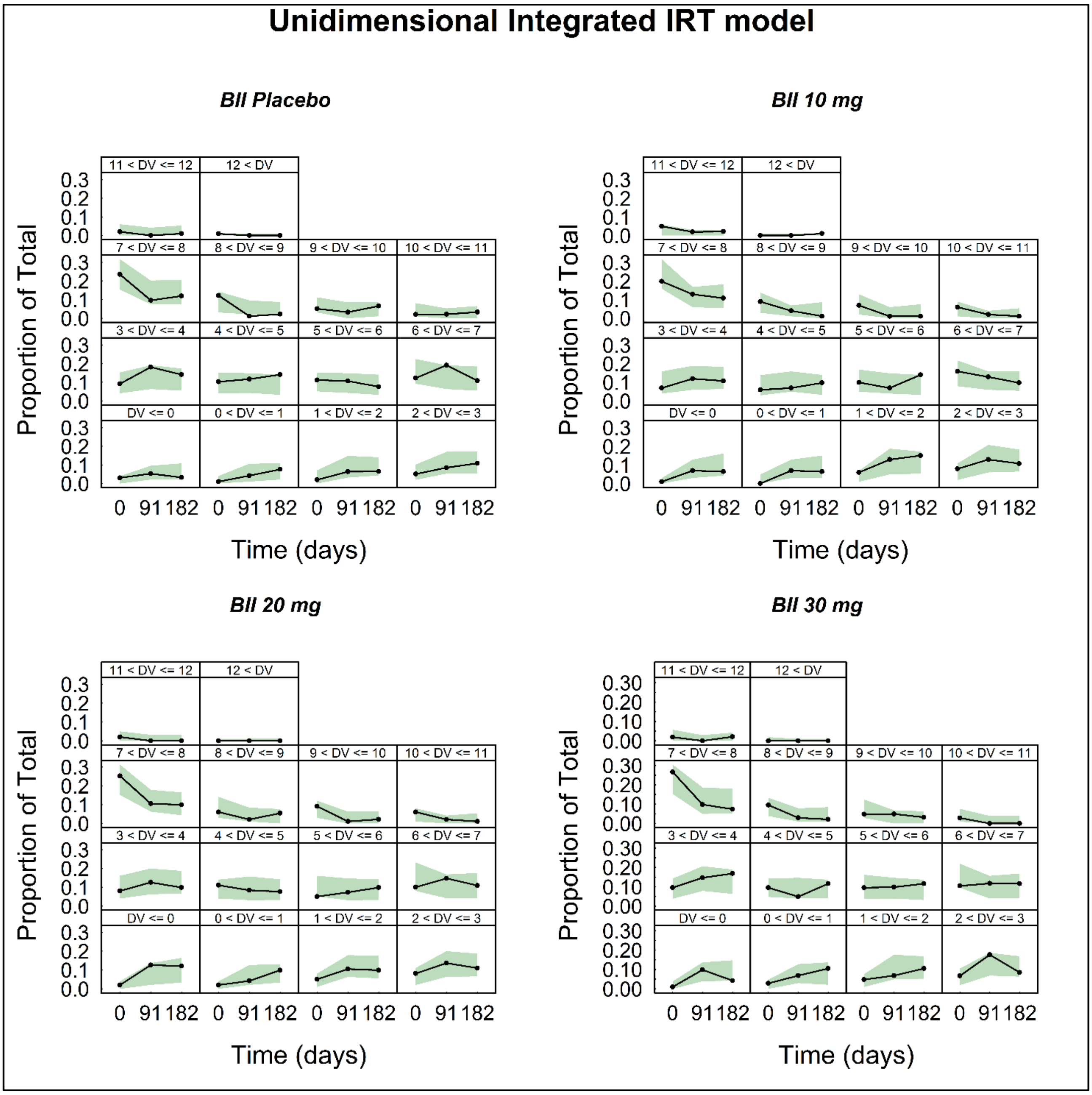

Figure S4.9 –Visual predictive check for the Benign Prostatic Hyperplasia Impact Index (BII) summary score in the unidimensional integrated item response theory (IRT) model stratified by treatment arm. The observed frequency of each score over time is shown as points and the shaded areas indicate the 95% confidence intervals of the frequencies of each score in 200 simulated datasets. DV: Dependent variable, i.e. observed score or score category (k).

5. Bidimensional Item response Theory Item parameter estimates

Table S5.1 – Item characteristic curve parameter estimates in the bidimensional integrated lower urinary tract symptoms due to benign prostatic hyperplasia (BPH-LUTS) item response theory model, with a compensatory model for the $BII_{summary}$. *a* denotes discrimination parameters while *b* denotes difficulty parameters for each score using the delta method (e.g. $B_{IPSS1,2} = b_{IPSS1,1} + b_{IPSS1,2}$). The first dimension comprises items IPSS1, IPSS3, IPSS5, and IPSS6, while the second dimension comprises IPSS2, IPSS4, IPSS7, and the QoL score. The BII item is associated with both dimensions in a compensatory fashion, and a discrimination parameter for each dimension is therefore reported (a_1 and a_2). Relative standard error was calculated as 100*(standard error of estimate/estimate). The typical value of η-shrinkage was was 9.3% and 10.1% in the first and second dimension, respectively. IPSS: International Prostate Symptom Score, QoL: Quality of Life, BII: BPH Impact Index.

Parameter	Estimate	Relative standard error
a_{IPSS1}	1.59	7.2%
$b_{IPSS1,1}$	-3.46	6.9%
$b_{IPSS1,2}$	1.59	7.7%
$b_{IPSS1,3}$	1.46	7.1%
$b_{IPSS1,4}$	1.22	7.2%
$b_{IPSS1,5}$	1.1	8.2%
a_{IPSS2}	1.13	9.6%
$b_{IPSS2,1}$	-5.73	8.7%
$b_{IPSS2,2}$	2.66	9.8%
$b_{IPSS2,3}$	2.13	9.2%
$b_{IPSS2,4}$	1.52	9.2%
$b_{IPSS2,5}$	1.52	9.5%
a_{IPSS3}	1.58	7.9%
$b_{IPSS3,1}$	-3.12	7.1%
$b_{IPSS3,2}$	1.53	7.8%
$b_{IPSS3,3}$	1.36	7.4%
$b_{IPSS3,4}$	0.905	7.9%
$b_{IPSS3,5}$	1.13	8.2%
a_{IPSS4}	0.893	9.9%
$b_{IPSS4,1}$	-4.42	8.6%
$b_{IPSS4,2}$	2.27	9.8%
$b_{IPSS4,3}$	1.87	9.5%
$b_{IPSS4,4}$	1.36	9.9%
$b_{IPSS4,5}$	1.56	10.3%
a_{IPSS5}	1.36	7.3%
$b_{IPSS5,1}$	-4.22	7.1%
$b_{IPSS5,2}$	1.94	8.0%
$b_{IPSS5,3}$	1.42	7.4%
$b_{IPSS5,4}$	1.1	7.4%

$b_{IPSS5,5}$	0.935	7.8%
a_{IPSS6}	1.23	7.9%
$b_{IPSS6,1}$	-2.49	7.2%
$b_{IPSS6,2}$	1.4	7.9%
$b_{IPSS6,3}$	1.37	7.7%
$b_{IPSS6,4}$	1.36	8.1%
$b_{IPSS6,5}$	1.34	9.7%
a_{IPSS7}	0.6	9.8%
$b_{IPSS7,1}$	-7.39	9.1%
$b_{IPSS7,2}$	4.56	10.0%
$b_{IPSS7,3}$	3.18	9.6%
$b_{IPSS7,4}$	2.16	10.1%
$b_{IPSS7,5}$	1.8	11.6%
a_{QoL}	1.34	9%
b_{QoL1}	-7.46	8.6%
$b_{QoL,2}$	2.85	10.9%
$b_{QoL,3}$	2.02	9.4%
$b_{QoL,4}$	1.92	8.9%
$b_{QoL,5}$	1.42	8.8%
$b_{QoL,6}$	1.61	9.4%
$a_{BII,Voiding}$	0.71	16.5%
$a_{BII,Storage}$	0.407	21.10%
$B_{BII,1}$	-4.99	4.1%
$B_{BII,2}$	0.941	12.4%
$B_{BII,3}$	0.909	9.7%
$B_{BII,4}$	0.797	8.7%
$B_{BII,5}$	0.747	8.2%
$B_{BII,6}$	0.531	9.3%
$B_{BII,7}$	0.535	9.2%
$B_{BII,8}$	0.766	7.9%
$B_{BII,9}$	1.25	6.9%
$B_{BII,10}$	0.668	12.5%
$B_{BII,11}$	0.89	14.0%
$B_{BII,12}$	1.01	18.2%
$B_{BII,13}$	2.42	28.4%
Post-baseline “voiding” disability variance	1.29	7.0%
Post-baseline “voiding” disability mean	-1.09	8.5%
Post-baseline “storage” disability variance	1.78	8.9%
Post-baseline “storage” disability mean	-1.67	8.4%
“Voiding” and “Storage” disability correlation	0.726	2.7%

6. Longitudinal bidimensional integrated item response theory model

6.1 Item-level visual predictive checks

6.1.1 International Prostate Symptom Score Item 1

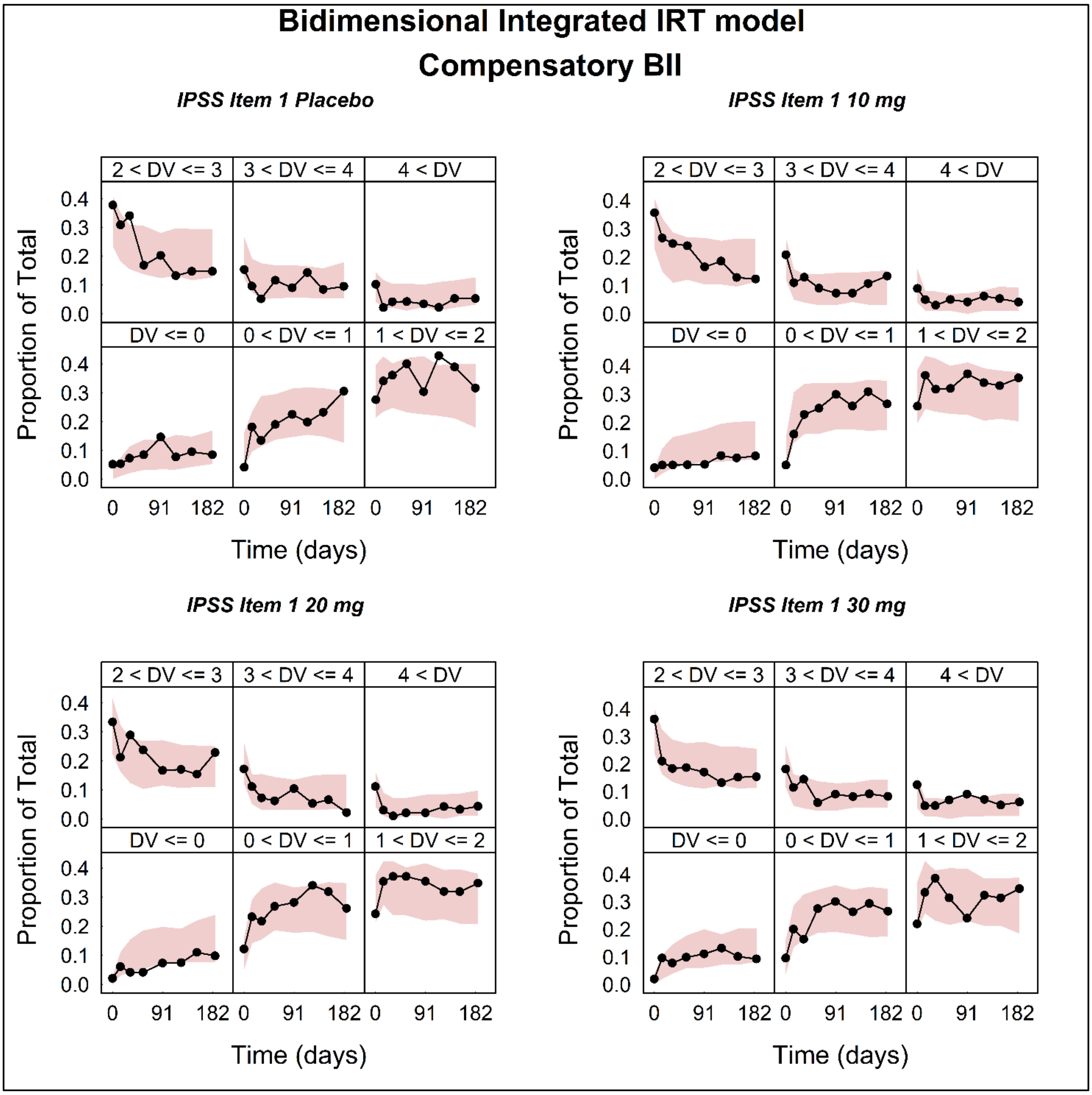

Figure S6.1 - Visual predictive check for the International Prostate Symptom Score (IPSS) item 1 “Incomplete Emptying” in the bidimensional integrated item response theory (IRT) model, which includes a compensatory model for the Benign Prostatic Hyperplasia Impact Index (BII) summary score, stratified by treatment arm. The observed frequency of each score over time is shown as points and the shaded areas

indicate the 95% confidence intervals of the frequencies of each score in 200 simulated datasets. DV: Dependent variable, i.e. observed score or score category (k).

6.1.2 International Prostate Symptom Score Item 2

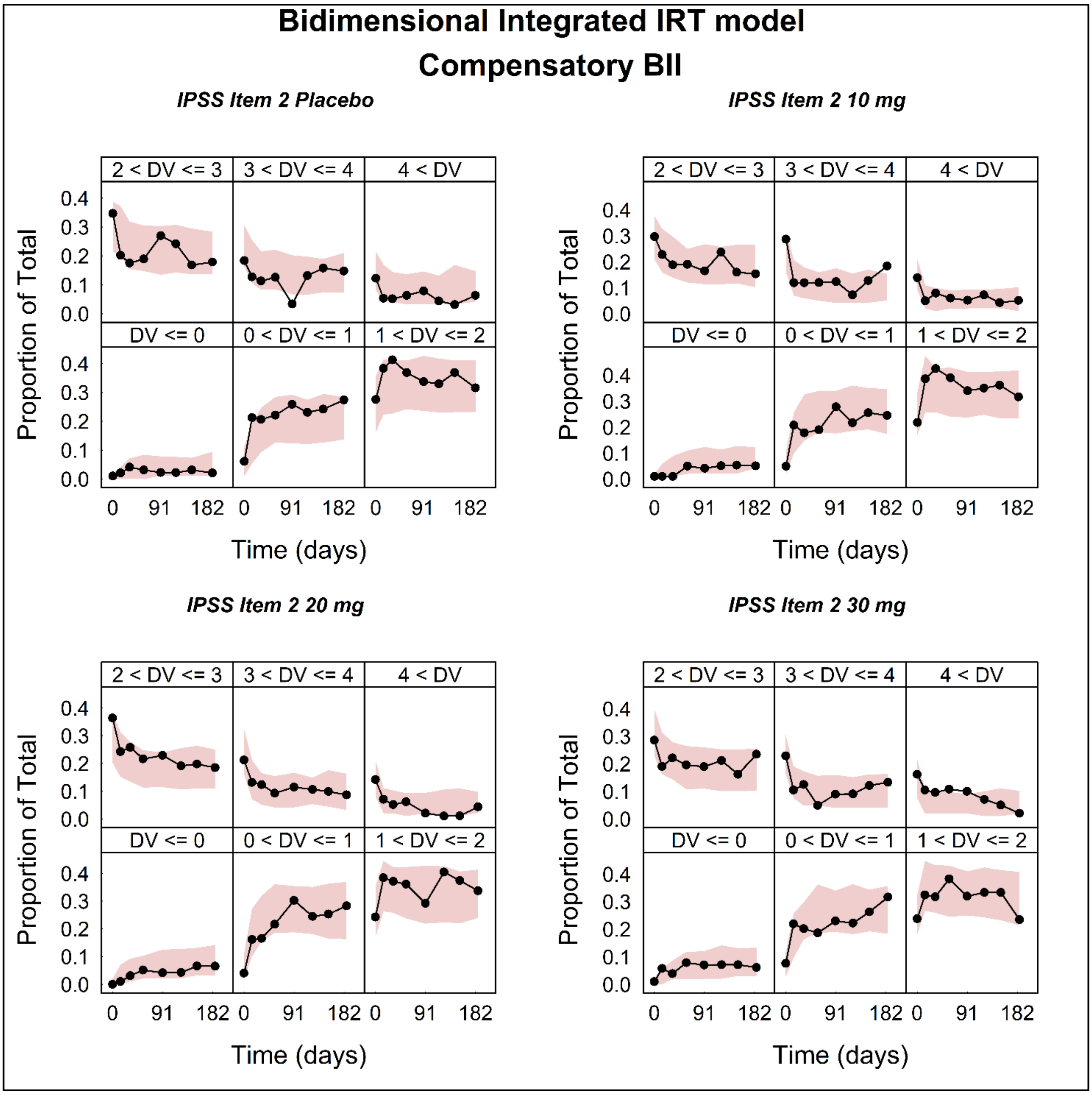

Figure S6.2 - Visual predictive check for the International Prostate Symptom Score (IPSS) item 2 "Frequency" in the bidimensional integrated item response theory (IRT) model, which includes a compensatory model for the Benign Prostatic Hyperplasia Impact Index (BII) summary score, stratified by treatment arm. The observed frequency of each score over time is shown as points and the shaded areas indicate the 95% confidence intervals of the frequencies of each score in 200 simulated datasets. DV: Dependent variable, i.e. observed score or score category (k).

6.1.3 International Prostate Symptom Score Item 3

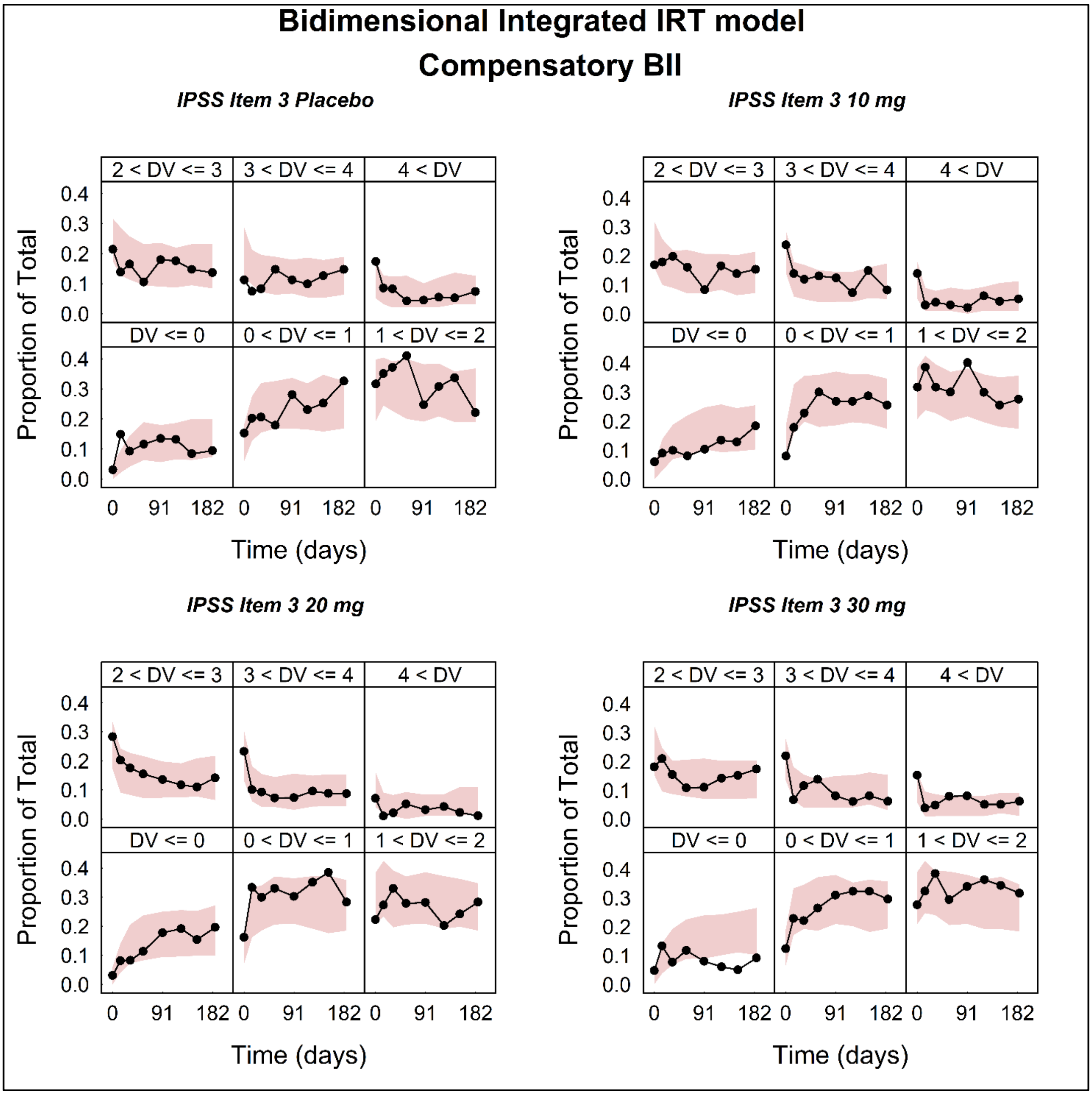

Figure S6.3 - Visual predictive check for the International Prostate Symptom Score (IPSS) item 3 "Intermittency" in the bidimensional integrated item response theory (IRT) model, which includes a compensatory model for the Benign Prostatic Hyperplasia Impact Index (BII) summary score, stratified by treatment arm. The observed frequency of each score over time is shown as points and the shaded areas indicate the 95% confidence intervals of the frequencies of each score in 200 simulated datasets. DV: Dependent variable, i.e. observed score or score category (k).

6.1.4 International Prostate Symptom Score Item 4

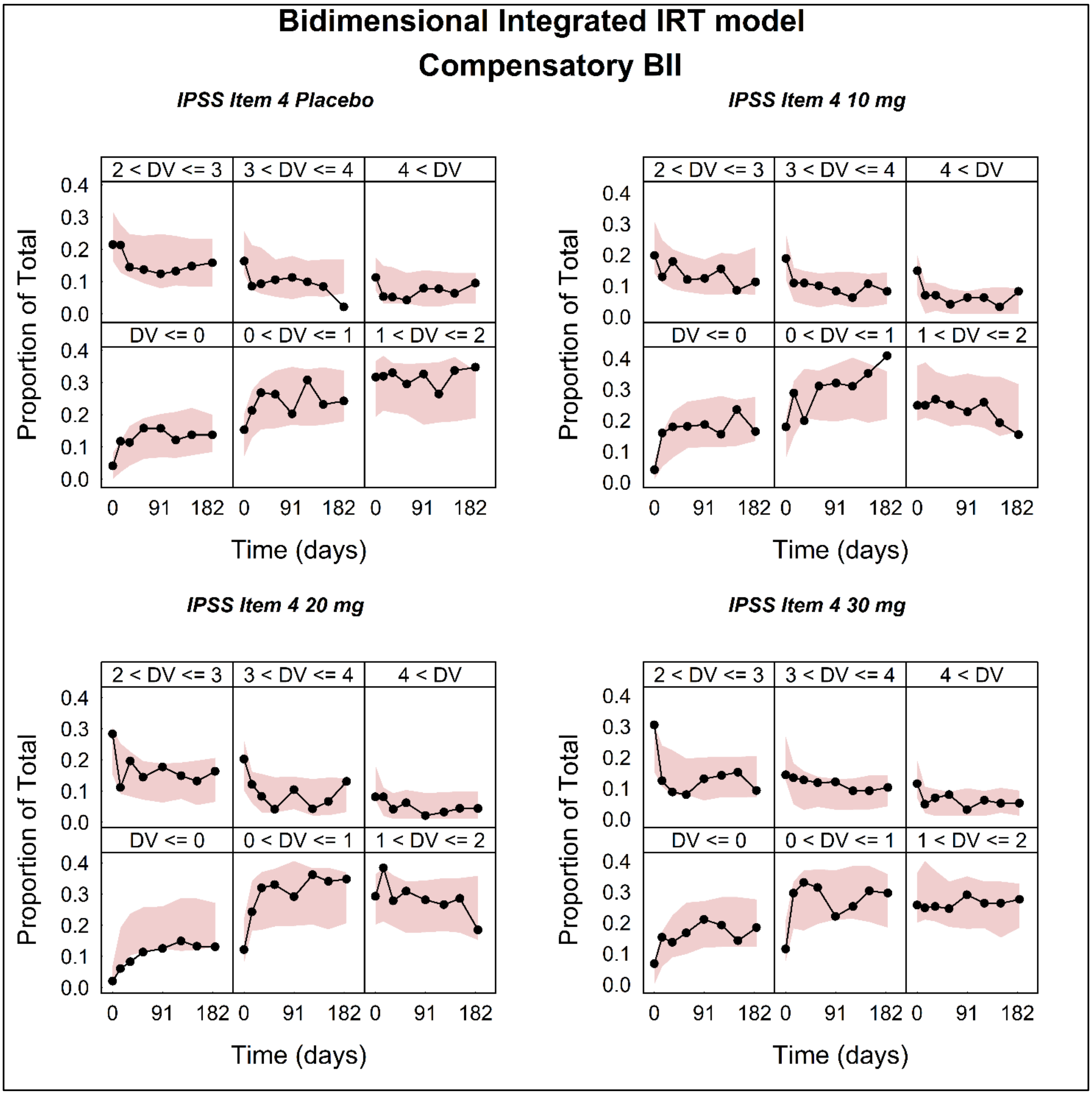

Figure S6.4 - Visual predictive check for the International Prostate Symptom Score (IPSS) item 4 “Urgency” in the bidimensional integrated item response theory (IRT) model, which includes a compensatory model for the Benign Prostatic Hyperplasia Impact Index (BII) summary score, stratified by treatment arm. The observed frequency of each score over time is shown as points and the shaded areas indicate the 95% confidence intervals of the frequencies of each score in 200 simulated datasets. DV: Dependent variable, i.e. observed score or score category (k).

6.1.5 International Prostate Symptom Score Item 5

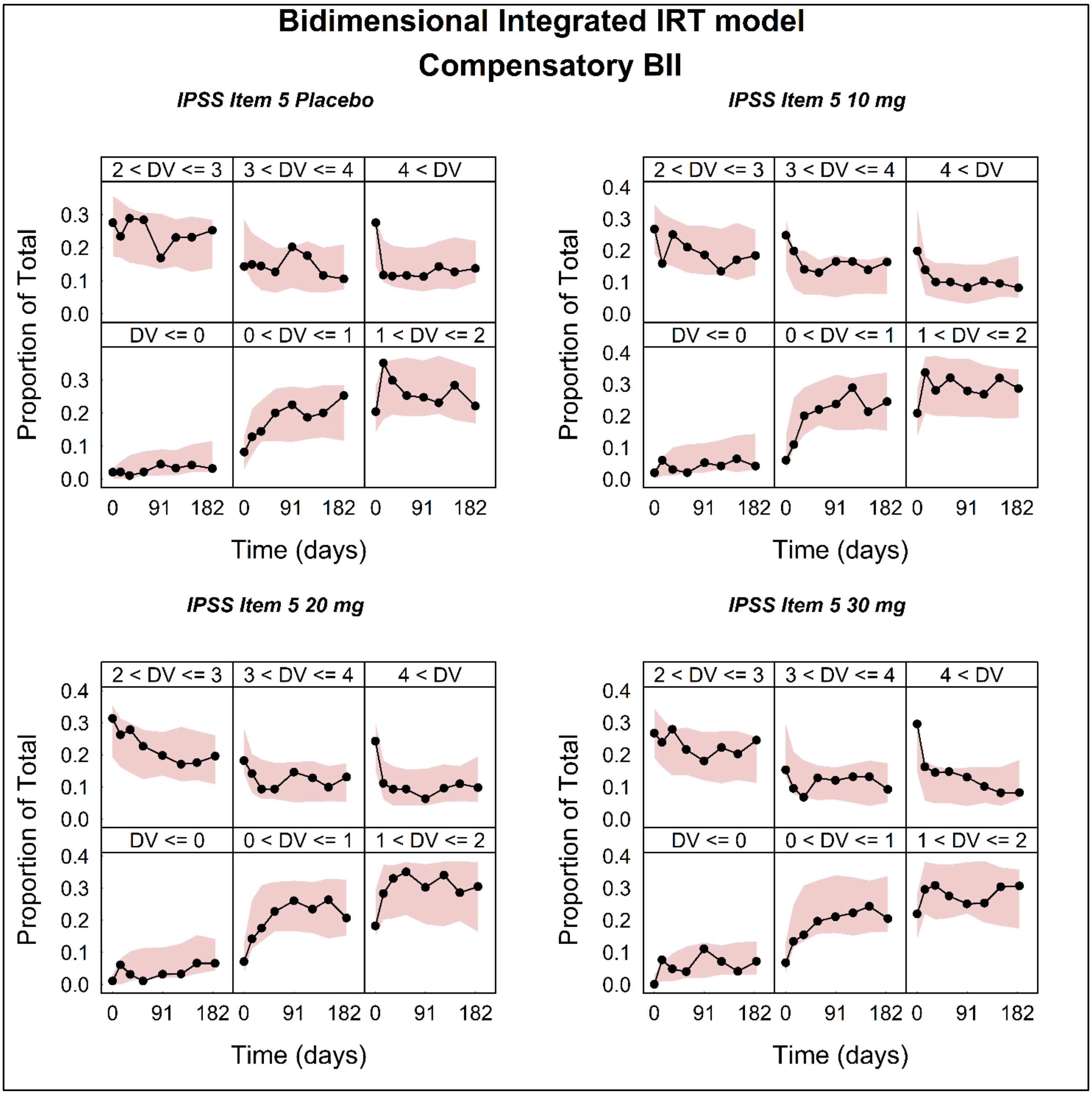

Figure S6.5 - Visual predictive check for the International Prostate Symptom Score (IPSS) item 5 “Weak Stream” in the bidimensional integrated item response theory (IRT) model, which includes a compensatory model for the Benign Prostatic Hyperplasia Impact Index (BII) summary score, stratified by treatment arm. The observed frequency of each score over time is shown as points and the shaded areas indicate the 95% confidence intervals of the frequencies of each score in 200 simulated datasets. DV: Dependent variable, i.e. observed score or score category (k).

6.1.6 International Prostate Symptom Score Item 6

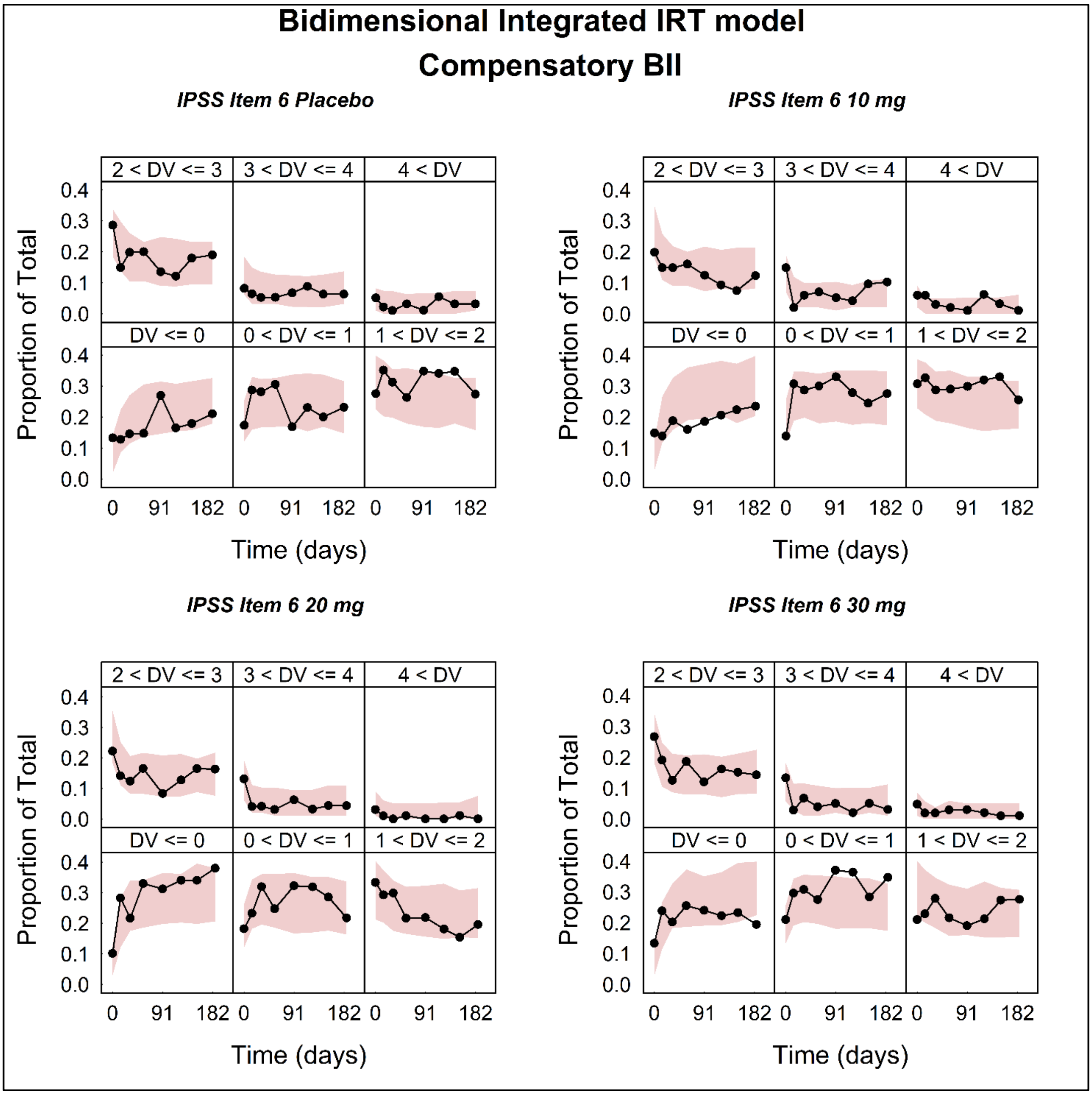

Figure S6.6 - Visual predictive check for the International Prostate Symptom Score (IPSS) item 6 "Straining" in the bidimensional integrated item response theory (IRT) model, which includes a compensatory model for the Benign Prostatic Hyperplasia Impact Index (BII) summary score, stratified by treatment arm. The observed frequency of each score over time is shown as points and the shaded areas indicate the 95% confidence intervals of the frequencies of each score in 200 simulated datasets. DV: Dependent variable, i.e. observed score or score category (k).

6.1.7 International Prostate Symptom Score Item 7

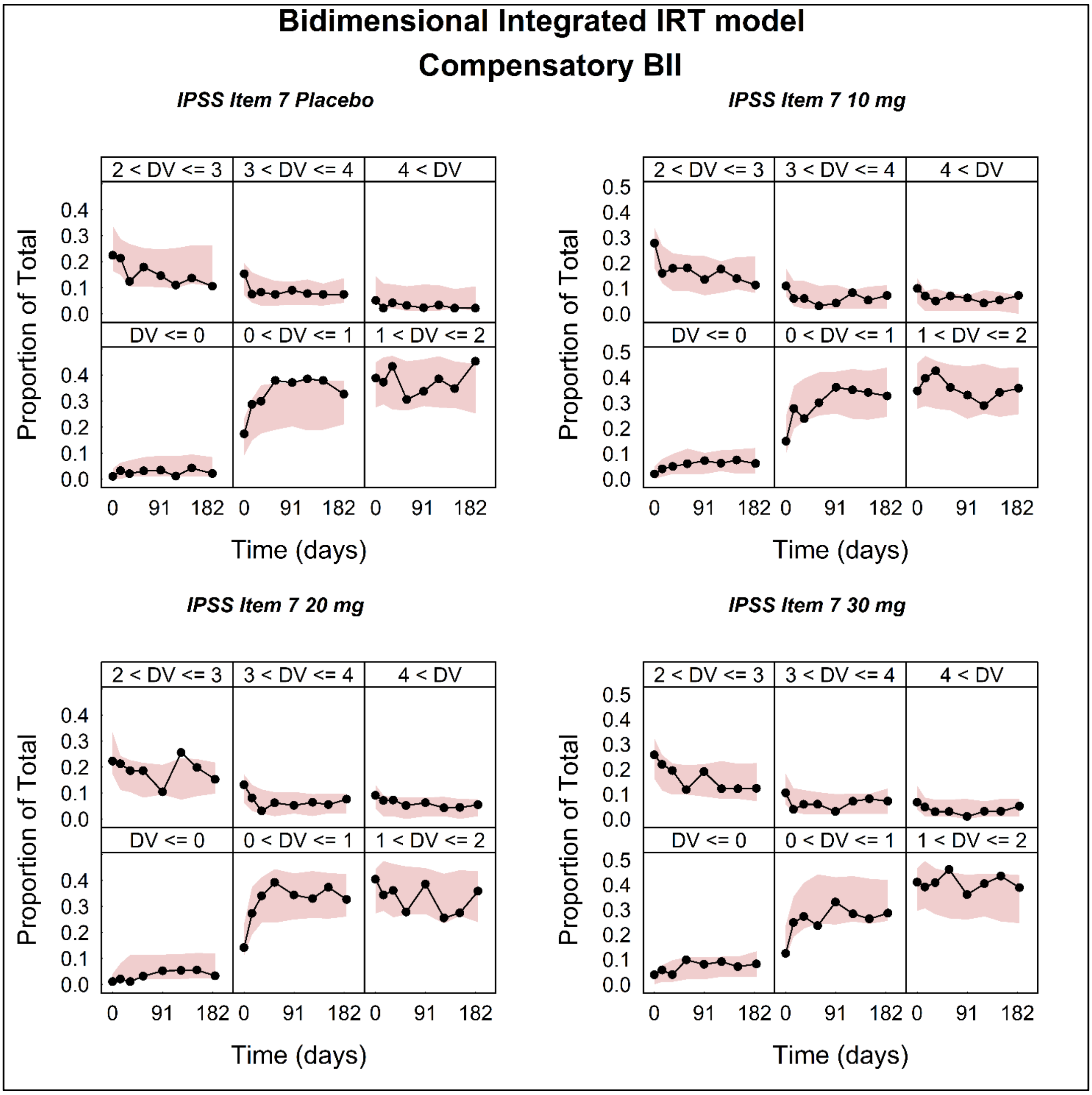

Figure S6.7 - Visual predictive check for the International Prostate Symptom Score (IPSS) item 7 “Nocturia” in the bidimensional integrated item response theory (IRT) model, which includes a compensatory model for the Benign Prostatic Hyperplasia Impact Index (BII) summary score, stratified by treatment arm. The observed frequency of each score over time is shown as points and the shaded areas indicate the 95% confidence intervals of the frequencies of each score in 200 simulated datasets. DV: Dependent variable, i.e. observed score or score category (k).

6.1.8 Quality of Life score

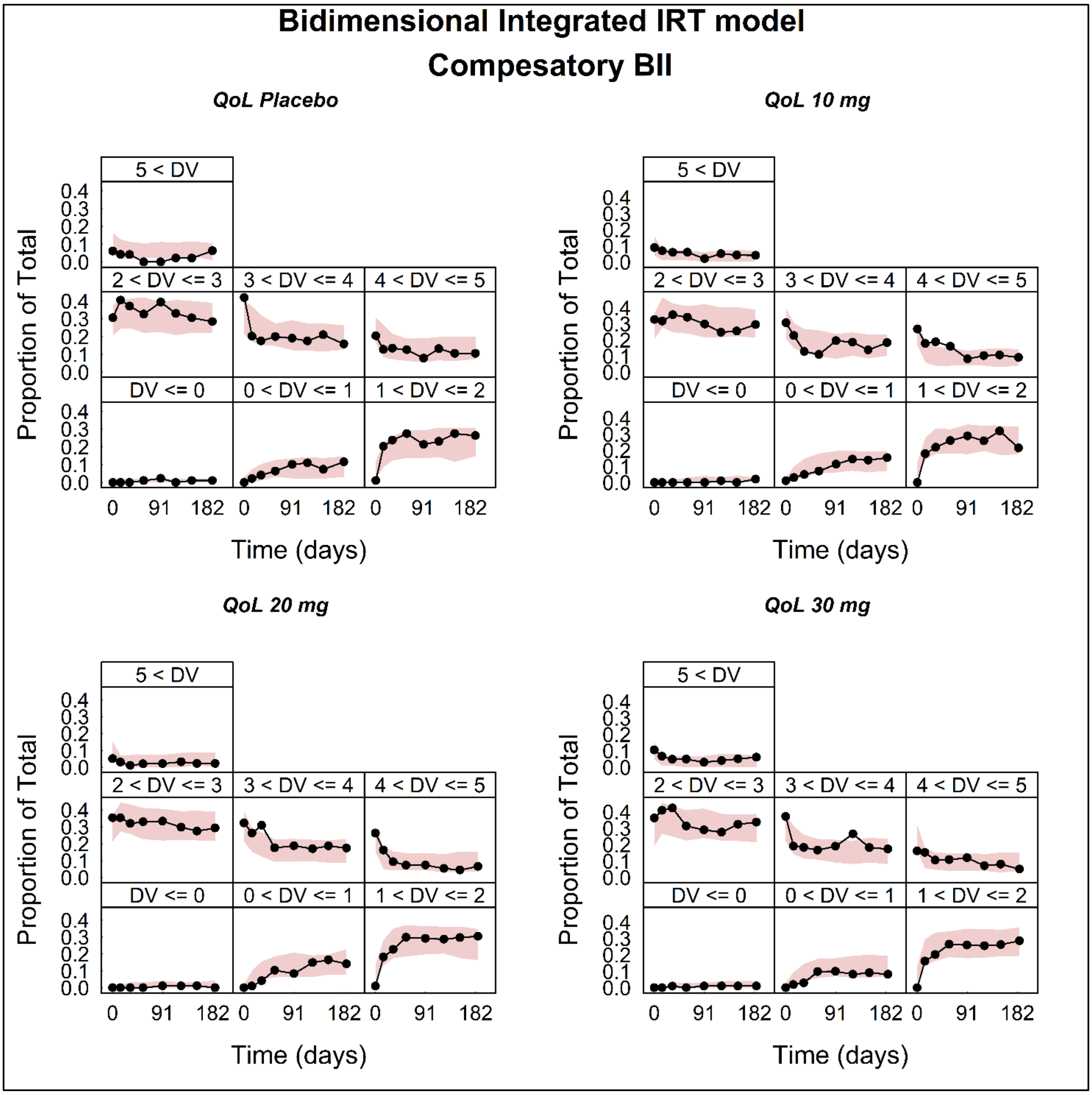

Figure S6.8 - Visual predictive check for the Quality of Life (QoL) score in the bidimensional integrated item response theory (IRT) model, which includes a compensatory model for the Benign Prostatic Hyperplasia Impact Index (BII) summary score, stratified by treatment arm. The observed frequency of each score over time is shown as points and the shaded areas indicate the 95% confidence intervals of the frequencies of each score in 200 simulated datasets. DV: Dependent variable, i.e. observed score or score category (k).

6.1.9 Benign Prostatic Hyperplasia Impact Index

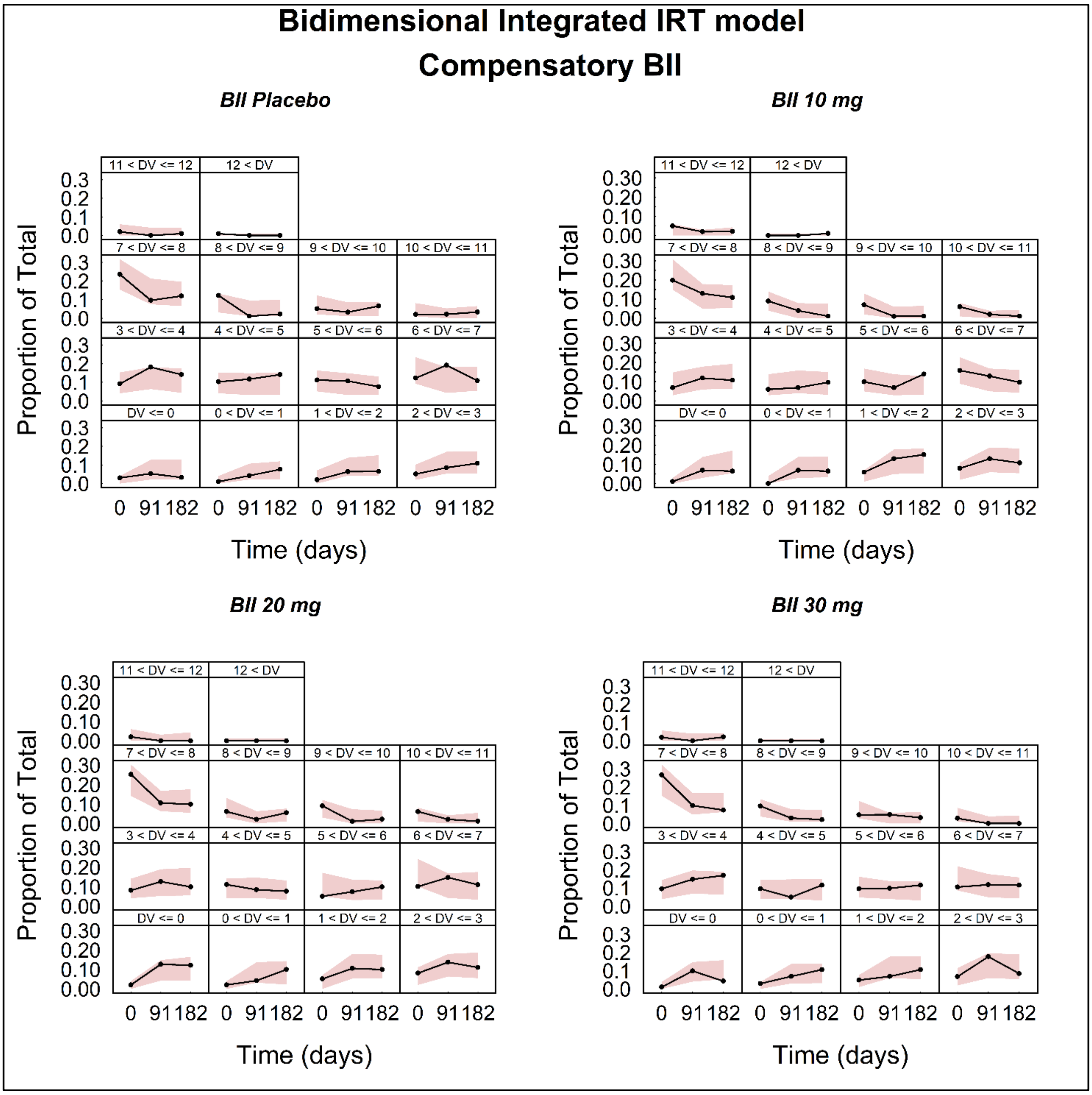

Figure S6.9 - Visual predictive check for the Benign Prostatic Hyperplasia Impact Index (BII) summary score in the bidimensional integrated item response theory (IRT) model, which includes a compensatory model for this item, stratified by treatment arm. The observed frequency of each score over time is shown as points and the shaded areas indicate the 95% confidence intervals of the frequencies of each score in 200 simulated datasets. DV: Dependent variable, i.e. observed score or score category (k).

7. Type I error in Stochastic Simulation and Estimation procedure

Table S7.1 – Type I error rate in the stochastic simulation and estimation procedure. 1,000 data sets were simulated from the reduced unidimensional integrated item response theory model (i.e. with no drug effect) at each sample size. Type I error rate was calculated as the number of times a significant drug effect was identified out of the total number of runs when estimating these data sets with the full model (i.e. with a drug effect).

**Model**	**Sample size**	**Actual type I error corresponding to a nominal type I error of 5% (95% CI)**
Unidimensional IPSS IRT	33	7.2% (5.4%; 9.0%)
Unidimensional Integrated IRT	33	7.1% (5.4%; 8.7%)
Unidimensional IPSS IRT	66	6.8% (5.1%; 8.3%)
Unidimensional Integrated IRT	66	7.1% (5.4%; 8.7%)
Unidimensional IPSS IRT	99	6.8% (5.2%; 8.4%)
Unidimensional Integrated IRT	99	5.6% (4.1%; 7.0%)
Unidimensional IPSS IRT	137	6.4% (4.9%; 7.9%)
Unidimensional Integrated IRT	137	6.9% (5.3%; 8.4%)
