# Supplementary material for: Integrated Item Response Theory Modeling of Multiple Patient-Reported Outcomes Assessing Lower Urinary Tract Symptoms Associated with Benign Prostatic Hyperplasia
Source: AAPS J. 2020 Jul 29;22(5):98. doi: 10.1208/s12248-020-00484-7 (PMC7391402; doi:10.1208/s12248-020-00484-7)
Supplement: Supplementary file 2 — (PDF 247 kb) [file 12248_2020_484_MOESM2_ESM.pdf]

```
$PROBLEM

$INPUT      ID ITEM NDAY TIME DV GRP MDV RESV

$DATA       ….csv IGNORE=@

$PRED

;Covariates ------------------------------------------------------------------------------------------------------------------------------

;;; BASERESV-DEFINITION START

IF(RESV.EQ.-99) THEN

   BASERESV = 0

ELSE

   BASERESV = ( 0 + THETA(71)*(RESV - 48.3))

ENDIF

;;; BASERESV-DEFINITION END

;;; BASE-RELATION START

BASECOV=BASERESV

;;; BASE-RELATION END

;Longitudinal parameters ------------------------------------------------------------------------------------------------------------------------------

TVBASE = THETA(66)

TVBASE = BASECOV+TVBASE

BASE = TVBASE + ETA(1) ; baseline latent disability

TVASY = THETA(67)

ASY=TVASY*(1+ETA(2)) ; Asymptote Maximum decrease in disease Status

TPROG = THETA(68)*EXP(ETA(4)) ; T1/2 describing disease progression rate

SLPREL=ETA(3) ; no theta associated with this parameter (i.e. fixed to 0)

TVDRUG=0

IF(TIME.GT.0.AND.GRP.GT.0) TVDRUG=THETA(70)

PSI = BASE + ASY*(1-EXP(-(LOG(2)/TPROG*TIME)**WEI)) + SLPREL*TIME + TVDRUG  ; longitudinal trajectory of latent disability

;--------------assignment of item parameters------------------------------------------

;Constants to select the model type

GR5=1

GR6=2

GR13=3

MODEL=0

;Define ICCs for IPSS items (ITEM=1 to ITEM=7)

IF(ITEM.EQ.1) THEN

                MODEL=GR5

                DIS=THETA(3) ; I1DISGR
```

```
        DIF1=THETA(4) ; I1DIF1GR
        DIF2=THETA(5) ; I1DIF2GR
        DIF3=THETA(6) ; I1DIF3GR
        DIF4=THETA(7) ; I1DIF4GR
        DIF5=THETA(8) ; I1DIF5GR
ENDIF
; excluded code for IPSS items 2 to 6 for brevity
IF(ITEM.EQ.7) THEN
        MODEL=GR5
        DIS=THETA(39) ; I7DISGR
        DIF1=THETA(40) ; I7DIF1GR
        DIF2=THETA(41) ; I7DIF2GR
        DIF3=THETA(42) ; I7DIF3GR
        DIF4=THETA(43) ; I7DIF4GR
        DIF5=THETA(44) ; I7DIF5GR
ENDIF
; Define QoL item
IF(ITEM.EQ.8) THEN
        MODEL=GR6
        DIS=THETA(45)  ; QoLDISGR
        DIF1=THETA(46) ; QoLDIF1GR
        DIF2=THETA(47) ; QoLDIF2GR
        DIF3=THETA(48) ; QoLDIF3GR
        DIF4=THETA(49) ; QoLDIF4GR
        DIF5=THETA(50) ; QoLDIF5GR
        DIF6=THETA(51) ; QoLDIF5GR
ENDIF
; Define BII summary score
IF(ITEM.EQ.9) THEN
        MODEL=GR13
        DIS=THETA(52)  ; BIIDISGR
        DIF1=THETA(53) ; BIIDIF1GR
        DIF2=THETA(54) ; BIIDIF2GR
        DIF3=THETA(55) ; BIIDIF3GR
        DIF4=THETA(56) ; BIIDIF4GR
        DIF5=THETA(57) ; BIIDIF5GR
```

```
        DIF6=THETA(58) ; BIIDIF6GR
        DIF7=THETA(59) ; BIIDIF7GR
        DIF8=THETA(60) ; BIIDIF8GR
        DIF9=THETA(61) ; BIIDIF9GR
        DIF10=THETA(62) ; BIIDIF10GR
        DIF11=THETA(63) ; BIIDIF11GR
        DIF12=THETA(64) ; BIIDIF12GR
        DIF13=THETA(65) ; BIIDIF13GR
ENDIF
;--------------------------IPSS Graded response model implementation (0-5) ----------------------------
IF(MODEL.EQ.GR5) THEN
DIFG1=DIF1
DIFG2=DIFG1+DIF2
DIFG3=DIFG2+DIF3
DIFG4=DIFG3+DIF4
DIFG5=DIFG4+DIF5
PGE1=EXP(DIS*(PSI-DIFG1))/(1+EXP(DIS*(PSI-DIFG1)))
PGE2=EXP(DIS*(PSI-DIFG2))/(1+EXP(DIS*(PSI-DIFG2)))
PGE3=EXP(DIS*(PSI-DIFG3))/(1+EXP(DIS*(PSI-DIFG3)))
PGE4=EXP(DIS*(PSI-DIFG4))/(1+EXP(DIS*(PSI-DIFG4)))
PGE5=EXP(DIS*(PSI-DIFG5))/(1+EXP(DIS*(PSI-DIFG5)))
P0=1-PGE1
P1=PGE1-PGE2
P2=PGE2-PGE3
P3=PGE3-PGE4
P4=PGE4-PGE5
P5=PGE5
ENDIF
;Select appropriate P(Y=k)
IF(MODEL.EQ.GR5.AND.DV.EQ.0) P=P0
IF(MODEL.EQ.GR5.AND.DV.EQ.1) P=P1
IF(MODEL.EQ.GR5.AND.DV.EQ.2) P=P2
IF(MODEL.EQ.GR5.AND.DV.EQ.3) P=P3
IF(MODEL.EQ.GR5.AND.DV.EQ.4) P=P4
IF(MODEL.EQ.GR5.AND.DV.EQ.5) P=P5
```

```
;IPRED = (P1*1) + (P2*2) + (P3*3) + (P4*4) + (P5*5)

;RES = DV - IPRED

;---------------------------QoL Graded response model implementation (0-6) -----------------------------

IF(MODEL.EQ.GR6) THEN

DIFG1=DIF1

DIFG2=DIFG1+DIF2

DIFG3=DIFG2+DIF3

DIFG4=DIFG3+DIF4

DIFG5=DIFG4+DIF5

DIFG6=DIFG5+DIF6

PGE1=EXP(DIS*(PSI-DIFG1))/(1+EXP(DIS*(PSI-DIFG1)))

;excluded PGE2 to PGE5 for brevity

PGE6=EXP(DIS*(PSI-DIFG6))/(1+EXP(DIS*(PSI-DIFG6)))

P0=1-PGE1

P1=PGE1-PGE2

,excluded P2 to P5 for brevity

P6=PGE6

ENDIF

;Select appropriate P(Y=k)

IF(MODEL.EQ.GR6.AND.DV.EQ.0) P=P0

;excluded P for DV=1 to DV=5 for brevity

IF(MODEL.EQ.GR6.AND.DV.EQ.6) P=P6

;--------------------------- BII Graded response model implementation (0-13) -----------------------------

IF(MODEL.EQ.GR13) THEN

DIFG1=DIF1

DIFG2=DIFG1+DIF2

;excluded DIFG3 TO DIFG12 for brevity

DIFG13=DIFG12+DIF13

PGE1=EXP(DIS*(PSI-DIFG1))/(1+EXP(DIS*(PSI-DIFG1)))

PGE2=EXP(DIS*(PSI-DIFG2))/(1+EXP(DIS*(PSI-DIFG2)))

;excluded PGE3 to PGE12 for brevity

PGE13=EXP(DIS*(PSI-DIFG13))/(1+EXP(DIS*(PSI-DIFG13)))

P0=1-PGE1

P1=PGE1-PGE2

;excluded P2 to P12 for brevity

P13=PGE13
```

```
ENDIF

Select appropriate P(Y=k)

IF(MODEL.EQ.GR13.AND.DV.EQ.0) P=P0

IF(MODEL.EQ.GR13.AND.DV.EQ.1) P=P1

;excluded definintion of P for DV=2 to DV=12

IF(MODEL.EQ.GR13.AND.DV.EQ.13) P=P13

;IPRED = (P1*1) + (P2*2) + (P3*3) + (P4*4) + (P5*5) + (P6*6) +(P7*7) + (P8*8) + (P9*9) + (P10*10) + (P11*11) + (P12*12) + (P13*13)

;RES = DV - IPRED

;------------------------Response probability prediction-------------------------

IF(P.LT.1E-16)     P = 1E-16     ; protection for P->0

IF(P.GT.(1-1E-16)) P = 1-1E-16   ; protection for P->1

Y=-2*LOG(P)

;--------------------------------Simulation code---------------------------------

;REP=IREP

;IF(ICALL.EQ.4) THEN

;               IF(MODEL.EQ.GR5) THEN

;               CALL RANDOM (2,R)

;               SDV=0

;               IF(R.LT.PGE1) SDV=1

;               IF(R.LT.PGE2) SDV=2

;               IF(R.LT.PGE3) SDV=3

;               IF(R.LT.PGE4) SDV=4

;               IF(R.LT.PGE5) SDV=5

;               ENDIF

;               IF(MODEL.EQ.GR6) THEN

;               CALL RANDOM (2,R)

;               SDV=0

;               IF(R.LT.PGE1) SDV=1

;               IF(R.LT.PGE2) SDV=2

;               IF(R.LT.PGE3) SDV=3

;               IF(R.LT.PGE4) SDV=4

;               IF(R.LT.PGE5) SDV=5

;               IF(R.LT.PGE6) SDV=6

;               ENDIF

;;              IF(MODEL.EQ.GR13) THEN

;               CALL RANDOM (2,R)
```

```
;               SDV=0
;               IF(R.LT.PGE1) SDV=1
;               IF(R.LT.PGE2) SDV=2
;               IF(R.LT.PGE3) SDV=3
;               IF(R.LT.PGE4) SDV=4
;               IF(R.LT.PGE5) SDV=5
;               IF(R.LT.PGE6) SDV=6
;               IF(R.LT.PGE7) SDV=7
;               IF(R.LT.PGE8) SDV=8
;               IF(R.LT.PGE9) SDV=9
;               IF(R.LT.PGE10) SDV=10
;               IF(R.LT.PGE11) SDV=11
;               IF(R.LT.PGE12) SDV=12
;               IF(R.LT.PGE13) SDV=13
;               ENDIF
;       DV=SDV
;ENDIF
;--------------------------estimation task ------------------------------------------

$ESTIMATION MAXEVAL=99999 METHOD=COND LAPLACE -2LL PRINT=1
$COVARIANCE PRINT=E
$THETA
(0) FIX ; theta not used
(0) FIX ; theta not used
; ICC parameters
(0, 1.19) FIX ; 3 I1DISGR
(-4.56) FIX ; 4 I1DIF1GR
(0, 2.02) FIX ; 5 I1DIF2GR
(0, 1.86) FIX ; 6 I1DIF3GR
(0, 1.57) FIX ; 7 I1DIF4GR
(0, 1.4) FIX ; 8 I1DIF5GR
(0, 1.04) FIX ; 9 I2DISGR
(-5.55) FIX ; 10 I2DIF1GR
(0, 2.65) FIX ; 11 I2DIF2GR
(0, 2.06) FIX ; 12 I2DIF3GR
(0, 1.49) FIX ; 13 I2DIF4GR
```

```
(0, 1.53) FIX ; 14 I2DIF5GR
;exlcluded thetas for items 3 to 9 for brevity
;longitudinal parameters
(-0.0372) ; 66 TVBASE
(-1.22) ; 67 TVASY
(0, 16.2) ; 68 TPROG
(0) FIX ; theta not used
(-0.567) ; 70 TVDRUG
(-20, 0.0025,20) ; 71 BASERESV1
$OMEGA  BLOCK(3)
 1.14
 0.205 1.78
 0 0.00495 0.000072
$OMEGA
 0.251
```
