# Supplementary material for: Integrated Item Response Theory Modeling of Multiple Patient-Reported Outcomes Assessing Lower Urinary Tract Symptoms Associated with Benign Prostatic Hyperplasia
Source: AAPS J. 2020 Jul 29;22(5):98. doi: 10.1208/s12248-020-00484-7 (PMC7391402; doi:10.1208/s12248-020-00484-7)
Supplement: Supplementary file 3 — (PDF 439 kb) [file 12248_2020_484_MOESM3_ESM.pdf]

; Bidimensional Integrated IRT model with compensatory graded response model for BII item

```
$PROBLEM

$INPUT ID ID2 OID ITEM NDAY TIME DV GRP MDV

$DATA ….csv IGNORE=@

$PRED

;longitudinal parameters

TVBASEV = THETA(67)

BASEV = TVBASEV + ETA(1)

TVBASES = THETA(68)

BASES = TVBASES + ETA(2)

TVASYV = THETA(69)

ASYV=TVASYV*(1+ETA(3)) ; Asymptote Maximum decrease in disease Status

TVASYS = THETA(70)

ASYS = TVASYS*(1+ETA(3)) ; Asymptote Maximum decrease in disease Status

TPROGV = THETA(71)*EXP(ETA(5)) ; T1/2 describing disease progression rate

TPROGS = THETA(72)*EXP(ETA(5)) ; T1/2 describing disease progression rate

SLPREL=ETA(4)

WEIV = THETA(73)

WEIS = THETA(73)

TVDRUGV=0

IF(TIME.GT.0.AND.GRP.GT.0) TVDRUGV=THETA(74)

TVDRUGS=0

IF(TIME.GT.0.AND.GRP.GT.0) TVDRUGS=THETA(75)

VOIDPSI = BASEV + ASYV*(1-EXP(-(LOG(2)/TPROGV*TIME)**WEIV)) + SLPREL*TIME + TVDRUGV

STORPSI = BASES + ASYS*(1-EXP(-(LOG(2)/TPROGS*TIME)**WEIS)) + SLPREL*TIME + TVDRUGS

IF(ITEM.EQ.1.OR.ITEM.EQ.3.OR.ITEM.EQ.5.OR.ITEM.EQ.6) PSI = VOIDPSI

IF(ITEM.EQ.2.OR.ITEM.EQ.4.OR.ITEM.EQ.7.OR.ITEM.EQ.8) PSI = STORPSI

; BII is defined in compensatory GR model

;--------------assignment of item parameters------------------------------------------

;Constants to select the model type

GR5=1

GR6=2

GR13=3

MODEL=0


;IPSS items
```

```
IF(ITEM.EQ.1) THEN
        MODEL=GR5
        DIS=THETA(3) ; I1DISGR
        DIF1=THETA(4) ; I1DIF1GR
        DIF2=THETA(5) ; I1DIF2GR
        DIF3=THETA(6) ; I1DIF3GR
        DIF4=THETA(7) ; I1DIF4GR
        DIF5=THETA(8) ; I1DIF5GR
ENDIF
; IPSS items 2 to 6 excluded for brevity
IF(ITEM.EQ.7) THEN
        MODEL=GR5
        DIS=THETA(39) ; I7DISGR
        DIF1=THETA(40) ; I7DIF1GR
        DIF2=THETA(41) ; I7DIF2GR
        DIF3=THETA(42) ; I7DIF3GR
        DIF4=THETA(43) ; I7DIF4GR
        DIF5=THETA(44) ; I7DIF5GR
ENDIF
; QoL item
IF(ITEM.EQ.8) THEN
        MODEL=GR6
        DIS=THETA(45)  ; QoLDISGR
        DIF1=THETA(46) ; QoLDIF1GR
        DIF2=THETA(47) ; QoLDIF2GR
        DIF3=THETA(48) ; QoLDIF3GR
        DIF4=THETA(49) ; QoLDIF4GR
        DIF5=THETA(50) ; QoLDIF5GR
        DIF6=THETA(51) ; QoLDIF5GR
ENDIF
; BII summary score
IF(ITEM.EQ.9) THEN
        MODEL=GR13
        DIS=THETA(52)  ; BIIDISGR latent variable 1
        DIS2 = THETA(66) ; BIIDISGR latent variable 2
```

```
        DIF1=THETA(53) ; BIIDIF1GR
        DIF2=THETA(54) ; BIIDIF2GR
        DIF3=THETA(55) ; BIIDIF3GR
        DIF4=THETA(56) ; BIIDIF4GR
        DIF5=THETA(57) ; BIIDIF5GR
        DIF6=THETA(58) ; BIIDIF6GR
        DIF7=THETA(59) ; BIIDIF7GR
        DIF8=THETA(60) ; BIIDIF8GR
        DIF9=THETA(61) ; BIIDIF9GR
        DIF10=THETA(62) ; BIIDIF10GR
        DIF11=THETA(63) ; BIIDIF11GR
        DIF12=THETA(64) ; BIIDIF12GR
        DIF13=THETA(65) ; BIIDIF13GR
ENDIF
;---------------------------IPSS Graded response model implementation (0-5) -----------------------------
IF(MODEL.EQ.GR5) THEN
DIFG1=DIF1
DIFG2=DIFG1+DIF2
DIFG3=DIFG2+DIF3
DIFG4=DIFG3+DIF4
DIFG5=DIFG4+DIF5
PGE1=EXP(DIS*(PSI-DIFG1))/(1+EXP(DIS*(PSI-DIFG1)))
PGE2=EXP(DIS*(PSI-DIFG2))/(1+EXP(DIS*(PSI-DIFG2)))
PGE3=EXP(DIS*(PSI-DIFG3))/(1+EXP(DIS*(PSI-DIFG3)))
PGE4=EXP(DIS*(PSI-DIFG4))/(1+EXP(DIS*(PSI-DIFG4)))
PGE5=EXP(DIS*(PSI-DIFG5))/(1+EXP(DIS*(PSI-DIFG5)))
P0=1-PGE1
P1=PGE1-PGE2
P2=PGE2-PGE3
P3=PGE3-PGE4
P4=PGE4-PGE5
P5=PGE5
ENDIF
;Select appropriate P(Y=k)
IF(MODEL.EQ.GR5.AND.DV.EQ.0) P=P0
IF(MODEL.EQ.GR5.AND.DV.EQ.1) P=P1
```

```
IF(MODEL.EQ.GR5.AND.DV.EQ.2) P=P2
IF(MODEL.EQ.GR5.AND.DV.EQ.3) P=P3
IF(MODEL.EQ.GR5.AND.DV.EQ.4) P=P4
IF(MODEL.EQ.GR5.AND.DV.EQ.5) P=P5
;IPRED = (P1*1) + (P2*2) + (P3*3) + (P4*4) + (P5*5)
;RES = DV - IPRED
;--------------------------QoL Graded response model implementation (0-6) ----------------------------
IF(MODEL.EQ.GR6) THEN
DIFG1=DIF1
DIFG2=DIFG1+DIF2
DIFG3=DIFG2+DIF3
DIFG4=DIFG3+DIF4
DIFG5=DIFG4+DIF5
DIFG6=DIFG5+DIF6
PGE1=EXP(DIS*(PSI-DIFG1))/(1+EXP(DIS*(PSI-DIFG1)))
PGE2=EXP(DIS*(PSI-DIFG2))/(1+EXP(DIS*(PSI-DIFG2)))
PGE3=EXP(DIS*(PSI-DIFG3))/(1+EXP(DIS*(PSI-DIFG3)))
PGE4=EXP(DIS*(PSI-DIFG4))/(1+EXP(DIS*(PSI-DIFG4)))
PGE5=EXP(DIS*(PSI-DIFG5))/(1+EXP(DIS*(PSI-DIFG5)))
PGE6=EXP(DIS*(PSI-DIFG6))/(1+EXP(DIS*(PSI-DIFG6)))
P0=1-PGE1
P1=PGE1-PGE2
P2=PGE2-PGE3
P3=PGE3-PGE4
P4=PGE4-PGE5
P5=PGE5-PGE6
P6=PGE6
ENDIF
;Select appropriate P(Y=k)
IF(MODEL.EQ.GR6.AND.DV.EQ.0) P=P0
IF(MODEL.EQ.GR6.AND.DV.EQ.1) P=P1
IF(MODEL.EQ.GR6.AND.DV.EQ.2) P=P2
IF(MODEL.EQ.GR6.AND.DV.EQ.3) P=P3
IF(MODEL.EQ.GR6.AND.DV.EQ.4) P=P4
IF(MODEL.EQ.GR6.AND.DV.EQ.5) P=P5
IF(MODEL.EQ.GR6.AND.DV.EQ.6) P=P6
```

```
;--------------------------- BII Graded response model implementation (0-13) -----------------------------
IF(MODEL.EQ.GR13) THEN
DIFG1=DIF1
DIFG2=DIFG1+DIF2
DIFG3=DIFG2+DIF3
DIFG4=DIFG3+DIF4
DIFG5=DIFG4+DIF5
DIFG6=DIFG5+DIF6
DIFG7=DIFG6+DIF7
DIFG8=DIFG7+DIF8
DIFG9=DIFG8+DIF9
DIFG10=DIFG9+DIF10
DIFG11=DIFG10+DIF11
DIFG12=DIFG11+DIF12
DIFG13=DIFG12+DIF13
PGE1=EXP(DIS*VOIDPSI+DIS2*STORPSI-DIFG1)  /  (1+EXP(DIS*VOIDPSI+DIS2*STORPSI-DIFG1))
PGE2=EXP(DIS*VOIDPSI+DIS2*STORPSI-DIFG2)  /  (1+EXP(DIS*VOIDPSI+DIS2*STORPSI-DIFG2))
PGE3=EXP(DIS*VOIDPSI+DIS2*STORPSI-DIFG3)  /  (1+EXP(DIS*VOIDPSI+DIS2*STORPSI-DIFG3))
PGE4=EXP(DIS*VOIDPSI+DIS2*STORPSI-DIFG4)  /  (1+EXP(DIS*VOIDPSI+DIS2*STORPSI-DIFG4))
PGE5=EXP(DIS*VOIDPSI+DIS2*STORPSI-DIFG5)  /  (1+EXP(DIS*VOIDPSI+DIS2*STORPSI-DIFG5))
PGE6=EXP(DIS*VOIDPSI+DIS2*STORPSI-DIFG6)  /  (1+EXP(DIS*VOIDPSI+DIS2*STORPSI-DIFG6))
PGE7=EXP(DIS*VOIDPSI+DIS2*STORPSI-DIFG7)  /  (1+EXP(DIS*VOIDPSI+DIS2*STORPSI-DIFG7))
PGE8=EXP(DIS*VOIDPSI+DIS2*STORPSI-DIFG8)  /  (1+EXP(DIS*VOIDPSI+DIS2*STORPSI-DIFG8))
PGE9=EXP(DIS*VOIDPSI+DIS2*STORPSI-DIFG9)  /  (1+EXP(DIS*VOIDPSI+DIS2*STORPSI-DIFG9))
PGE10=EXP(DIS*VOIDPSI+DIS2*STORPSI-DIFG10)  /  (1+EXP(DIS*VOIDPSI+DIS2*STORPSI-DIFG10))
PGE11=EXP(DIS*VOIDPSI+DIS2*STORPSI-DIFG11)  /  (1+EXP(DIS*VOIDPSI+DIS2*STORPSI-DIFG11))
PGE12=EXP(DIS*VOIDPSI+DIS2*STORPSI-DIFG12)  /  (1+EXP(DIS*VOIDPSI+DIS2*STORPSI-DIFG12))
PGE13=EXP(DIS*VOIDPSI+DIS2*STORPSI-DIFG13)  /  (1+EXP(DIS*VOIDPSI+DIS2*STORPSI-DIFG13))
P0=1-PGE1
P1=PGE1-PGE2
P2=PGE2-PGE3
P3=PGE3-PGE4
P4=PGE4-PGE5
P5=PGE5-PGE6
P6=PGE6-PGE7
P7=PGE7-PGE8
```

```
P8=PGE8-PGE9

P9=PGE9-PGE10

P10=PGE10-PGE11

P11=PGE11-PGE12

P12=PGE12-PGE13

P13=PGE13

ENDIF

;Select appropriate P(Y=k)

IF(MODEL.EQ.GR13.AND.DV.EQ.0) P=P0

IF(MODEL.EQ.GR13.AND.DV.EQ.1) P=P1

IF(MODEL.EQ.GR13.AND.DV.EQ.2) P=P2

IF(MODEL.EQ.GR13.AND.DV.EQ.3) P=P3

IF(MODEL.EQ.GR13.AND.DV.EQ.4) P=P4

IF(MODEL.EQ.GR13.AND.DV.EQ.5) P=P5

IF(MODEL.EQ.GR13.AND.DV.EQ.6) P=P6

IF(MODEL.EQ.GR13.AND.DV.EQ.7) P=P7

IF(MODEL.EQ.GR13.AND.DV.EQ.8) P=P8

IF(MODEL.EQ.GR13.AND.DV.EQ.9) P=P9

IF(MODEL.EQ.GR13.AND.DV.EQ.10) P=P10

IF(MODEL.EQ.GR13.AND.DV.EQ.11) P=P11

IF(MODEL.EQ.GR13.AND.DV.EQ.12) P=P12

IF(MODEL.EQ.GR13.AND.DV.EQ.13) P=P13

;IPRED = (P1*1) + (P2*2) + (P3*3) + (P4*4) + (P5*5) + (P6*6) +(P7*7) + (P8*8) + (P9*9) + (P10*10) + (P11*11) + (P12*12) + (P13*13)

;RES = DV - IPRED

;------------------------Response probability prediction-------------------------

IF(P.LT.1E-16)     P = 1E-16     ; protection for P->0

IF(P.GT.(1-1E-16)) P = 1-1E-16   ; protection for P->1

Y=-2*LOG(P)

;-------------------------------Simulation code--------------------------------

;IF(ICALL.EQ.4) THEN

;           CALL RANDOM (2,R)

;           SDV=0

;           IF(R.LT.PGE1) SDV=1

;           IF(R.LT.PGE2) SDV=2

;           IF(R.LT.PGE3) SDV=3

;           IF(R.LT.PGE4) SDV=4
```

```
;               IF(R.LT.PGE5) SDV=5
;
;               IF(ITEM.EQ.1) SCORE=0
;               IF(ITEM.EQ.100) DV=SCORE
;               SCORE=SCORE+SDV
;               DV=SDV
;ENDIF
;---------------------------estimation task -------------------------------------------
$COV PRINT=E
$EST MAXEVAL=99999 METHOD=COND LAPLACE -2LL PRINT=1 NUMERICAL NSIG=2 MCETA=10 NOABORT MSFO=msf_run7778
$TABLE ID NDAY TIME ITEM DV PSI MDV FILE=psi_estimates_tab7778 NOAPPEND ONEHEADER NOPRINT
$TABLE ITEM DIS DIF1 DIF2 DIF3 DIF4 DIF5 DIF6 DIF7 DIF8 DIF9 DIF10 DIF11 DIF12 DIF13 MDV FILE=item_parameters_tab7778 NOAPPEND
ONEHEADER NOPRINT
$TABLE ID TIME ETA1 ETA2 PSI FILE=param_estimates_tab7778 NOAPPEND ONEHEADER NOPRINT
;---------------------------fixed effects for slope and baseline-----------------------
$THETA
(0) FIX ;
(0) FIX ;
(0, 1.59) FIX ; 3 I1DISGR
(-3.46) FIX ; 4 I1DIF1GR
(0, 1.59) FIX ; 5 I1DIF2GR
(0, 1.46) FIX ; 6 I1DIF3GR
(0, 1.22) FIX ; 7 I1DIF4GR
(0, 1.1) FIX ; 8 I1DIF5GR
(0, 1.13) FIX ; 9 I2DISGR
(-5.73) FIX ; 10 I2DIF1GR
(0, 2.66) FIX ; 11 I2DIF2GR
(0, 2.13) FIX ; 12 I2DIF3GR
(0, 1.52) FIX ; 13 I2DIF4GR
(0, 1.52) FIX ; 14 I2DIF5GR
(0, 1.58) FIX ; 15 I3DISGR
(-3.12) FIX ; 16 I3DIF1GR
(0, 1.53) FIX ; 17 I3DIF2GR
(0, 1.36) FIX ; 18 I3DIF3GR
(0, 0.905) FIX ; 19 I3DIF4GR
(0, 1.13) FIX ; 20 I3DIF5GR
(0, 0.893) FIX ; 21 I4DISGR
```

(-4.42) FIX ; 22 I4DIF1GR

(0, 2.27) FIX ; 23 I4DIF2GR

(0, 1.87) FIX ; 24 I4DIF3GR

(0, 1.36) FIX ; 25 I4DIF4GR

(0, 1.56) FIX ; 26 I4DIF5GR

(0, 1.36) FIX ; 27 I5DISGR

(-4.22) FIX ; 28 I5DIF1GR

(0, 1.94) FIX ; 29 I5DIF2GR

(0, 1.42) FIX ; 30 I5DIF3GR

(0, 1.1) FIX ; 31 I5DIF4GR

(0, 0.935) FIX ; 32 I5DIF5GR

(0, 1.23) FIX ; 33 I6DISGR

(-2.49) FIX ; 34 I6DIF1GR

(0, 1.4) FIX ; 35 I6DIF2GR

(0, 1.37) FIX ; 36 I6DIF3GR

(0, 1.36) FIX ; 37 I6DIF4GR

(0, 1.34) FIX ; 38 I6DIF5GR

(0, 0.6) FIX ; 39 I7DISGR

(-7.39) FIX ; 40 I7DIF1GR

(0, 4.56) FIX ; 41 I7DIF2GR

(0, 3.18) FIX ; 42 I7DIF3GR

(0, 2.16) FIX ; 43 I7DIF4GR

(0, 1.8) FIX ; 44 I7DIF5GR

(0, 1.34) FIX ; 45 QoLDISGR

(-7.46) FIX ; 46 QoLDIF1GR

(0, 2.85) FIX ; 47 QoLDIF2GR

(0, 2.02) FIX ; 48 QoLDIF3GR

(0, 1.92) FIX ; 49 QoLDIF4GR

(0, 1.42) FIX ; 50 QoLDIF5GR

(0, 1.61) FIX ; 51 QoLDIF6GR

(0, 0.71) FIX ; 52 BIIDISGR

(-4.99) FIX ; 53 BIIDIF1GR

(0, 0.941) FIX ; 54 BIIDIF2GR

(0, 0.909) FIX ; 55 BIIDIF3GR

(0, 0.797) FIX ; 56 BIIDIF4GR

(0, 0.747) FIX ; 57 BIIDIF5GR

```
(0, 0.531) FIX ; 58 BIIDIF6GR
(0, 0.535) FIX ; 59 BIIDIF7GR
(0, 0.766) FIX ; 60 BIIDIF8GR
(0, 1.25) FIX ; 61 BIIDIF9GR
(0, 0.668) FIX ; 62 BIIDIF10GR
(0, 0.89) FIX ; 63 BIIDIF11GR
(0, 1.01) FIX ; 64 BIIDIF12GR
(0, 2.42) FIX ; 65 BIIDIF13GR
(0, 0.407) FIX ; 66 DIS2 BII
(-0.0398) ; 67 BASEV
(-0.0718) ; 68 BASES
(-0.762) ; 69 ASYV
(-0.972) ; 70 ASYS
(0, 15.2) ; 71 TPROGV
(0, 11) ; 72 TPROGS
(0, 1.41) ; 73 wei
(-0.55) ; 74 DRUGV
(-0.827) ; 75 DRUGS
$OMEGA  BLOCK(2)
 1.03  ; IIV BASEV
 0.418 1.65  ; IIV BASES
 $OMEGA BLOCK(2)
1.53  ; IIV ASYV
0.00297 0.000045  ; SLPREL
 $OMEGA
 0.314 ; TPROG
```
